# Barriers to and Facilitators of Using eHealth to Support Gestational Diabetes Mellitus Self-management: Systematic Literature Review of Perceptions of Health Care Professionals and Women With Gestational Diabetes Mellitus

**DOI:** 10.2196/39689

**Published:** 2022-10-27

**Authors:** Ladan Safiee, Daniel John Rough, Heather Whitford

**Affiliations:** 1 School of Science and Engineering University of Dundee Dundee United Kingdom; 2 School of Health Sciences University of Dundee Dundee United Kingdom

**Keywords:** gestational diabetes mellitus, GDM, gestational diabetes, self-management, eHealth

## Abstract

**Background:**

Gestational diabetes mellitus (GDM) is one of the most common medical complications during pregnancy. eHealth technologies are proving to be successful in supporting the self-management of medical conditions. Digital technologies have the potential to improve GDM self-management.

**Objective:**

The primary objective of this systematic literature review was to identify the views of health professionals (HPs) and women with GDM regarding the use of eHealth for GDM self-management. The secondary objective was to investigate the usability and user satisfaction levels when using these technologies.

**Methods:**

Following the PRISMA (Preferred Reporting Items for Systematic Reviews and Meta-Analyses) approach, the search included primary papers in English on the evaluation of technology to support self-management of GDM from January 2008 to September 2021 using MEDLINE, CINAHL, Embase, ACM, and IEEE databases. The lists of references from previous systematic literature reviews, which were related to technology and GDM, were also examined for primary studies. Papers with qualitative, quantitative, and mixed methodologies were included and evaluated. The selected papers were assessed for quality using the Cochrane Collaboration tool, National Institute for Health and Care Excellence clinical guidelines, Critical Appraisal Skills Programme Qualitative Checklist, and McGill University Mixed Methods Appraisal Tool. NVivo (QSR International) was used to extract qualitative data, which were subjected to thematic analysis. Narrative synthesis was used to analyze the quantitative data.

**Results:**

A total of 26 papers were included in the review. Of these, 19% (5/26) of studies used quantitative research methodologies, 19% (5/26) used qualitative methods, and 62% (16/26) used mixed methods. In all, 4 themes were identified from the qualitative data: the benefits of using technology, engagement with people via technology, the usability of technology, and discouragement factors for the use of technology. The thematic analysis revealed a vast scope of challenges and facilitators in the use of GDM self-management systems. The challenges included usability aspects of the system, technical problems, data privacy, lack of emotional support, the accuracy of reported data, and adoption of the system by HPs. Convenience, improved GDM self-management, peer support, increased motivation, increased independence, and consistent monitoring were facilitators to use these technologies. Quantitative data showed that there is potential for improving the usability of the GDM self-management systems. It also showed that convenience, usefulness, increasing motivation for GDM self-management, helping with GDM self-management, and being monitored by HPs were facilitators to use the GDM self-management systems.

**Conclusions:**

This novel systematic literature review shows that HPs and women with GDM encountered some challenges in using GDM self-management systems. The usability of GDM systems was the primary challenge derived from qualitative and quantitative results, with convenience, consistent monitoring, and optimization of GDM self-management emerging as important facilitators.

## Introduction

### Background

Gestational diabetes mellitus (GDM) is defined as any degree of carbohydrate intolerance with onset or first recognition during pregnancy [1]. GDM is one of the most common medical complications of pregnancy [2], with a significant increase in its prevalence in different ethnic groups and countries over the last several years [3,4]. GDM is most prevalent in the Middle East and North Africa, with an estimated median of 12.9%, and least prevalent in Europe, with an estimated median of 5.8% of all pregnancies [5]. In the United Kingdom, the prevalence of GDM is approximately 4% of all pregnancies [6]. The rate of GDM is likely to rise owing to a growth in GDM risk factors, such as greater prevalence of maternal obesity and advancing age of childbearing [7], leading to an increasing demand for GDM clinical services [8].

GDM is associated with serious maternal [9-11] and fetal complications [12-15]. Mothers who have been affected by GDM are also at risk of developing type 2 diabetes [16] and cardiometabolic disorders later in life [17], and their infants are more at risk of developing adulthood obesity and type 2 diabetes [12,18]. These complications represent significant health problems and cost [19] for health services. The risk of adverse effects of GDM can be minimized by good control over maternal blood glucose (BG), diet, and physical activities [20]. However, there is limited time between diagnosis and delivery to optimize care for women with GDM [21]. Therefore, regular clinic visits [22] to a multidisciplinary team are advised to provide care during pregnancy. Nonetheless, traveling to specialist clinics in central locations [23] is expensive [24], time consuming, and inconvenient for women [25]. Recently, there has been an increase in the use of technology to enable self-management of GDM by women and to shift GDM management away from hospital-based care [26].

In light of increased adoption of technology to access information and communication, a digital GDM self-management system might offer advantages such as reducing patient travel and waiting time [27], saving medical practitioner time [8], reducing costs [28,29] to both the health care system and patients, greater convenience [30], attainment of better pregnancy outcomes [31], and an increased feeling of self-efficacy [32]. This can further lead to better BG control [29,33] and a decrease in GDM complications owing to greater accuracy and more frequent monitoring [34]. Such outcomes are evident in the results of several studies, which have found that health care technology can be beneficial for women with GDM in the improvement of hemoglobin A_1c_ [35-37], mean BG [21,38-40], maternal weight [41], and maternal and fetal outcomes [38,42,43]. Technology could also offer high-quality remote health care in a critical situation such as the COVID-19 pandemic to women with GDM, where travel and in-person contact have been severely restricted [44,45]. Therefore, there is an urgent need to consider computer-based communication technologies for the management of diabetes. This could contribute to better diabetes management by improving patient knowledge, attitudes, skills, lifestyle behavior [46], quality of care, and access to care [29].

### Study Aims

Digital GDM self-management systems developed in recent years are available mostly as mobile apps or websites [8,30,34] and offer a wide range of features such as monitoring BG [23], diet, physical activity, blood pressure, and ketonuria [8] for women with GDM. However, a recent study by Kalhori et al [47] suggests that the few GDM apps available in popular app stores are poor in quality, using the Mobile App Rating Scale as a basis for this result [47].

Furthermore, most GDM self-management systems are not widely used [48,49], and some are no longer supported [8,50], one reason for which is obsolete hardware (ML Bartholomew, MD, email communication, 2018). Previous systematic reviews in the scope of technology and GDM management were carried out on available technology for GDM self-management [47,51-53], the impact of technology on clinical and pregnancy outcomes or GDM management [54-56], comparing women’s clinical outcomes using technology with standard care [35], and the psychological aspect of using technology [57]. However, to the best of our knowledge, there is no systematic literature review of the opinions of health care professionals and women with GDM about using technology for GDM self-management.

The primary aim of this systematic literature review was to identify the views of health professionals (HPs) and women with GDM regarding barriers and facilitators of using technology for GDM self-management. The secondary aim was to investigate the usability and user satisfaction of these technologies.

## Methods

### Approach

The search strategy was developed by following the PRISMA (Preferred Reporting Items for Systematic Reviews and Meta-Analyses) approach [58] with the help of a professional librarian. The PRISMA guidelines lead to standardized reports and enhance the clarity of systematic literature reviews [59].

### Criteria of Inclusion and Exclusion

To achieve the aims of this review, the criteria for inclusion and exclusion were developed as presented in Textbox 1.

**Inclusion and exclusion criteria.**

**Inclusion criteria**

1. Views of health care professionals, pregnant women diagnosed with gestational diabetes mellitus (GDM) or postpartum women with a history of GDM about their pregnancy period
2. Technology (eHealth or telemedicine being used, evaluated, reviewed, or discussed by participants) or usability evaluation or reports of user satisfaction levels
3. Any primary research studies
4. Aspects of GDM management (eg, blood glucose control, diet, weight, physical activity, medication adherence, or information)

**Exclusion criteria**

1. Published papers written in any language other than English
2. Women with preexisting type 1 and type 2 diabetes (except papers that provide information about GDM distinct from type 1 and 2 diabetes)
3. Any nondigital technology
4. Papers published before 2008
5. Posters, abstracts, and news items
6. Systematic literature reviews
7. Usability results for task performance

### Search Strategy and Screening Process

A search was carried out using 3 search terms—“self-management,” “gestational diabetes,” and “technology” (Multimedia Appendix 1). The search terms were identified from papers in eHealth for GDM in the PubMed database.

The search included publications written in English from January 2008 to September 2021 in the MEDLINE, CINAHL, Embase, ACM, and IEEE databases. This date limitation was chosen to represent contemporary technology for GDM self-management.

The screening process was conducted by the first author in line with previous studies [60,61] and with the help of the research team and a professional librarian using the following steps:

1. Identification: the results of the search from different databases were exported to the EndNote X7 software. Furthermore, the reference lists of previous systematic literature reviews related to technology and GDM were examined in the primary studies. All citations were collated into one group and duplicate records were removed.
2. Screening: the titles and abstracts of the remaining citations were screened based on the inclusion and exclusion criteria to select potential papers by the first author. At this stage, 2 other members of the research team independently conducted a double screening of the first 10% of the results. Following a discussion phase, this screening process was repeated to ensure reliability based on inclusion and exclusion criteria.
3. Eligibility: Mendeley software was used to keep electronic copies of the full text of potential papers. The full text of the papers was assessed based on the inclusion and exclusion criteria.
4. Included: the final papers were selected from the full text based on the inclusion and exclusion criteria by the first author. The papers were discussed with the research team if there was any lack of clarity in their inclusion.

### Data Extraction

The study characteristics were extracted from the final 26 included papers. A predefined data extraction table was populated with information, such as study design, sample size, location, analysis method, participants’ ages, inclusion and exclusion criteria, analysis methods, study goals, quantitative and qualitative data collection tools, and key findings (Multimedia Appendix 2 [13, 21, 25, 27, 30, 34, 41, 43, 48, 50, 62-77]).

NVivo 12 was used to extract relevant qualitative data to achieve the primary aim of the review. A predefined table, including the author, measures, scale items, and results, was used to extract relevant quantitative data.

### Quality Assessment

Appropriate appraisal tools were chosen based on the methodology and study design. Each of the studies included in this review was critically assessed using an appropriate tool: the Cochrane Collaboration tool for randomized controlled trials (RCTs) [78], National Institute for Health and Care Excellence clinical guidelines for questionnaire studies or surveys [79], the Critical Appraisal Skills Programme Qualitative Checklist for qualitative studies [80], and the McGill University Appraisal Tool for Mixed Methods [81].

To meet the aims of this systematic literature review and not to exclude data relevant to this review, the quality of papers was not assessed with the purpose of excluding them. Instead, limitations of the included studies were considered during the analysis and synthesis of data.

### Analysis

The analysis was completed in 2 phases for qualitative and quantitative data. Thematic analysis with an inductive approach [82] was used to develop themes from 73% (19/26) studies that included qualitative data following the 6 steps outlined by Braun and Clarke [82].

Level 1 (reviewing codes of each theme for existence of coherent patterns) and level 2 analyses (reviewing the themes to assess whether they reflect the entire data set) were conducted by the first author and the second coauthor. Interrater reliability was not carried out, in line with the recommended process by Braun and Clarke [83].

Narrative review was used to analyze the quantitative data owing to the heterogeneity of research methods used. A narrative review is flexible and allows different types of evidence to be combined into a coherent summary. The narrative review process [84] included summarizing and explaining the quantitative data presented in 69% (18/26) included papers.

## Results

### Study Selection and Study Characteristics

**Figure 1:**
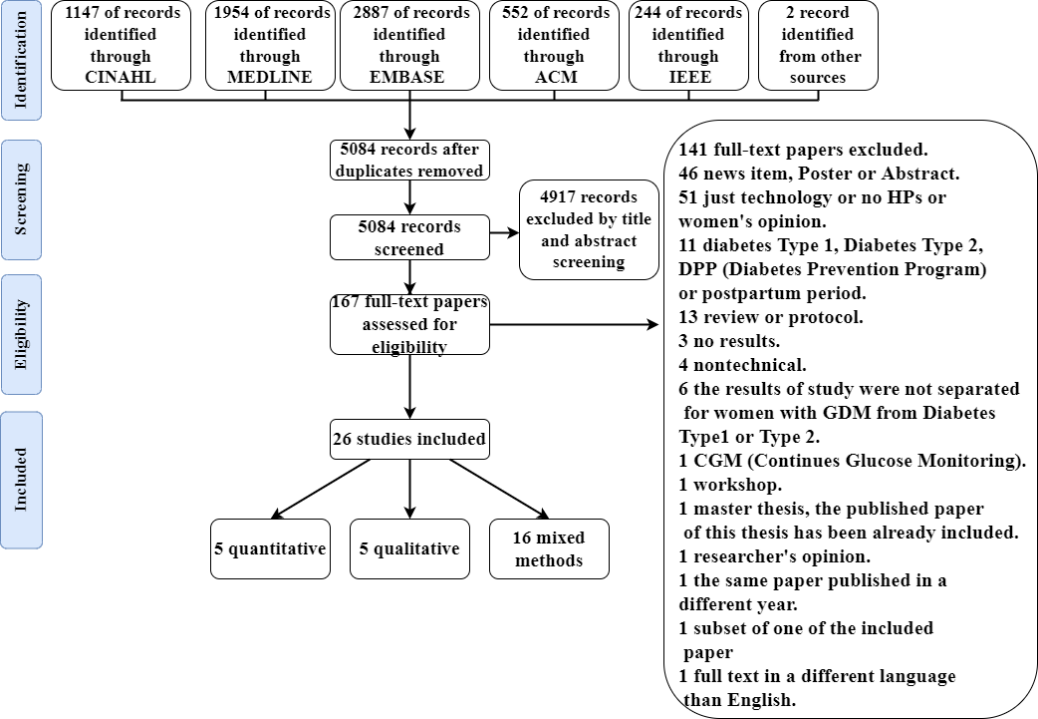
Study identification flowchart. GDM: gestational diabetes mellitus; HP: health professional.

The search and screening strategies are shown in Figure 1.

A total of 26 papers were included from the full text based on the inclusion and exclusion criteria. Of the included papers, 19% (5/26) were quantitative, 19% (5/26) were qualitative, and 62% (16/26) used mixed methods (Multimedia Appendix 2). The sample sizes varied among the studies, ranging from 9 [62] to 340 [63] participants. Most of the included studies were from Europe (15/26, 58%), and the rest were from North America (3/26, 11%), Australia (4/26, 15%), Singapore (1/26, 4%), New Zealand (1/26, 4%), and South Korea (1/26, 4%), with 4% (1/26) study of unspecified location. Studies varied in exploring the views of women and HPs. Of these, 96% (25/26) studies included the views of women, with 23% (6/26) including the views of HPs, and only 4% (1/26) including HPs’ views without those of women.

### Methodological Quality Assessment

In general, the 26 included studies showed some degree of bias in their research.

**Figure 2:**
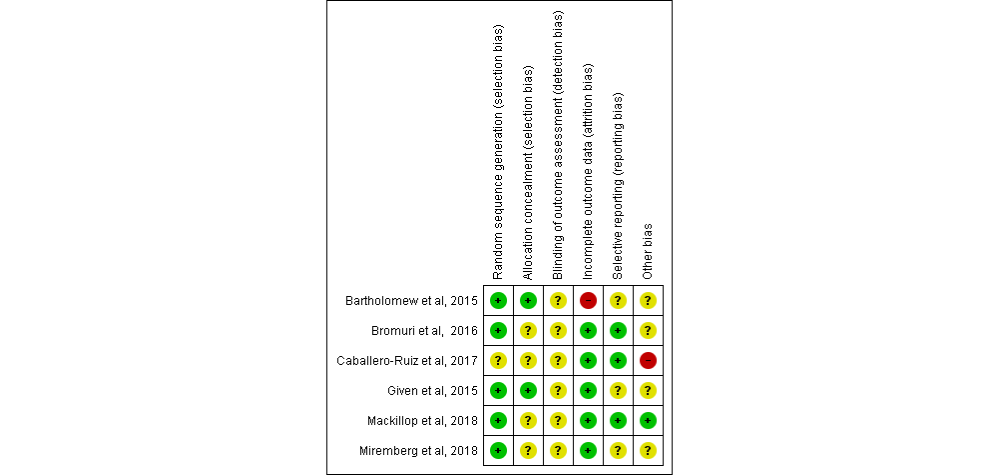
Risk of bias summary—each risk of bias item across included randomized controlled trial studies. Green: Yes (low risk of bias); Red: No (high risk of bias); Yellow: Unclear (bias is not clear or bias cannot be determined) [21,25,34,43,50,64].

**Figure 3:**
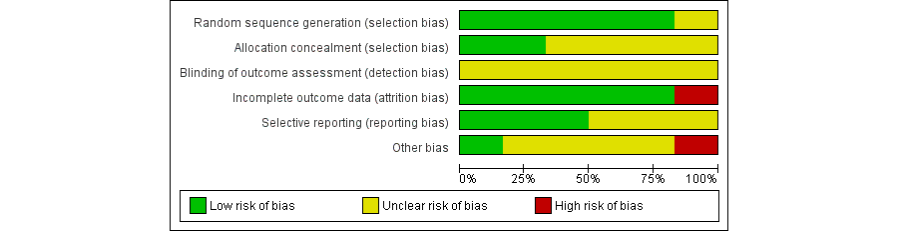
Risk of bias graph—the risk of bias item presented as percentages across included randomized controlled trial studies. Green: Yes (low risk of bias); Red: No (high risk of bias); Yellow=Unclear (bias is not clear or bias cannot be determined).

Figure 2 [21,25,34,43,50,64] and Figure 3 show the risk of bias summary and graph (specific to an RCT study design), respectively, for the included studies using Review Manager 5.3 (Cochrane Collaboration desktop software).

On the basis of the nature of the included studies that used technology as a core of their research, it was impossible to blind participants and researchers from the knowledge of the intervention participants received [35]. Therefore, performance bias was not included in the risk of bias assessment (Figures 2 and 3) [35]. Of the 23% (6/26) RCT studies, quality assessment showed that 15% (4/26) had a low risk of bias [21,25,43,64]. The other 8% (2/26) studies presented a risk of bias in incomplete outcome data owing to the withdrawal of a large number of participants during the study [50] and an unequal number of participants in the intervention and control groups [34]. Furthermore, the allocation concealment method has been adequately reported in only 8% (2/26) studies [25,50].

Quality appraisal of the remaining studies (Multimedia Appendix 3 [13,21,25,27,30,34,41,43,48,50,62-77]) revealed that 11% (3/26) qualitative studies were of good quality in design, data collection procedure, and data analysis [62,65,66]. The common limitations for the rest of the studies (including quantitative, qualitative, or mixed methods) were bias in sampling [49,67,68], small sample sizes relative to the type of study conducted [67,69,70], lack of information about the validity and reliability of the data collection tools [67,69-71], lack of information about inclusion and exclusion criteria [68], poor qualitative results [64], and unclear recruitment strategy [72]. In addition, there was a lack of information regarding the method of gathering qualitative data [13] and the analysis process [13,72]. In 8% (2/26) mixed methods studies, it was stated that the quantitative data would be collected in the following phase, but there was no clear explanation about how the triangulation of the quantitative and qualitative data would answer the research question [27,48].

### Thematic Analysis of Qualitative Data

#### Overview

**Figure 4:**
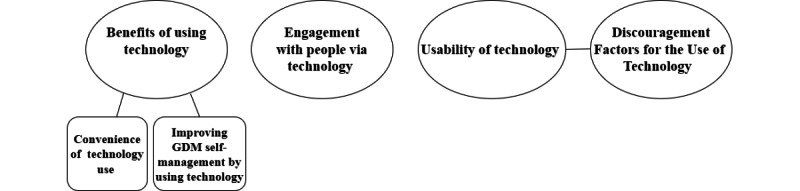
Thematic map showing themes and subthemes. GDM: gestational diabetes mellitus.

Of the included studies, 73% (19/26) contributed qualitative data to the thematic analysis. The views of women and HPs were integrated and reported together throughout the analysis. A total of 4 themes were identified: benefits of using technology, engagement with people via technology, usability of technology, and discouragement factors for the use of technology (definitions of the themes and subthemes are available in Multimedia Appendix 4). Furthermore, 2 subthemes were identified, as outlined in Figure 4.

#### Theme 1: Benefits of Using Technology

##### Overview

Both women and HPs reported their confidence in [27,72] and willingness to use GDM self-management systems because of the benefits of these systems for women with GDM [25,30] and for their babies’ health [65,66,72]. Furthermore, some HPs considered technology to be beneficial for complementing the limited number of health care professionals, while the rate of GDM is increasing [66]. The benefits of using technology themes included 2 subthemes: “convenience of technology use” and “improving self-management by using technology.”

##### Convenience of Technology Use

Convenience was the predominant benefit of using technology for GDM management. A total of 50% (13/26) papers reported that women with GDM and HPs found the convenience of reduced travel and clinical appointments, as well as the pervasive use of technology, the most beneficial reasons for its use. Women in the studies of Khalil [66] and Edward et al [73] expressed that traveling is “exhausting” [66] particularly toward the end of their pregnancy [73], and especially for women living at a distance [27,30,66]. Women and HPs also indicated that it would lead to a reduction in the need for women to make potentially stressful arrangements for finding childcare and managing absence from work [25,27]. Therefore, technology could be highly advantageous for women with busy lives, especially those who already have children [30]:

I am amazed with the technology and it suited me much better than having to travel in a lot and wait, especially with little ones

Patient 10

Generally, women and HPs lauded the ease and convenience of using technology rather than traditional paper logbooks. This was mainly because of the ability to access technology anytime [74] or anywhere, driven by the growing pervasiveness of mobile devices [62,73]—“you’ve always got your phone haven’t you, so it’s the easiest way to do stuff” (Patient 3).

Women and HPs also recognized constant access to information related to GDM [62,65,73] and being familiar with using similar technology [48,62,72-74] as further elements of ease and convenience.

Saving time is another convenient aspect of technology use for both women [25,27,75] and HPs [64]. In a study by Bromuri et al [64], a telemedicine system helped HPs review BG values quicker than to review them on a paper logbook, owing to alerts that highlighted out-of-range BG values resulting in hyperglycemia and hypoglycemia being recognized quickly. Women and HPs agreed that it takes considerable time to attend clinical appointments [27] just to “be told you’re doing everything right” [25,75]:

They don’t want to spend all of their time trying to get to the hospital and look for parking and spend long periods waiting at hospital.

Clinician 2

##### Improving GDM Self-management by Using Technology

Improving the ability of women to self-manage GDM is another prominent benefit of using such technology. Increasing awareness of one’s own data has been perceived as an important element of using technology for GDM self-management [25,63,65]. Women in some studies indicated that real-time feedback [63,65,74], visualization of data (eg, graphic nutrient summaries or recommendations) [63,65,74] and the ability to review and track their data [25,63,75] empowered them with “self-awareness” about their own data [63,65,74]. The clarity of the relationship between different attributes, particularly diet and BG levels, was seen as beneficial [63,65,73]. Data relationships also helped women to identify *“*where it [self-management] was working or where it was going wrong*’’* [25] and supported them to change their lifestyle [63,65,74]. However, women and HPs had different opinions about the accuracy of women’s self-reported data. Although some women favored recording data with technology because they were more accurate and precise [74], other women admitted misreporting their data values to get more positive feedback [65]. Some HPs did not want to rely on women’s self-reported data [76] because they did not trust the accuracy of the data; they preferred to enter data into the system themselves [72].

Women also found information related to diet [49,63,68,73,74] and peer support [73,75] useful in improving their lifestyle. Moreover, women felt “automatic messages” [63], rewards, and goal tracking on the system motivated them to change their lifestyle and optimize their GDM self-management.

Both women and HPs perceived increased independence through technology [66,72]. Women and HPs also expressed that using a digital GDM system improved both their self-management skills [27,65,66] and exercise of control on their GDM condition [25,27,63,65,71,73,74,77]:

myDiabby helped patients self-manage their health. [66]

Nurse 2

Technologies help us being more autonomous. We feel more responsible. [66]

Patient 1

#### Theme 2: Engagement With People via Technology

This theme included 2 main components including engagement with peers and engagement with health care professionals.

Women with GDM indicated that accessing “peer support” by a digital GDM self-management system would be useful [75] as “somebody may know something more” [68]. Although some women had little or no experience with web-based group communication, they were still interested in communicating with other women with GDM via technology [68]. Peer support provided an opportunity for women to access “other people’s experiences” [73] for sharing and exchanging information [68]. As a woman with GDM indicated, peer support provided “a better overview of risks associated with GDM, what could go wrong potentially, and the good stories as well” [73]. Overall, women perceived that peer support empowered them with a broader scope of GDM knowledge than other women who were experiencing the same condition [73]. In addition, peer support reassured women that they were “not alone” [73] and offered them a “constant feeling of support” [73]. Furthermore, it enabled women to talk about their condition and experience in a “safe space” without being judged by other people [73]. Women indicated a lack of peer support in the current care system that might be addressed using technology [75].

Regarding engagement with health care professionals, women appreciated the possibility of receiving additional support using technology. They valued sharing their data and having regular GDM monitoring by HPs via technology [25,66,68,72,73,76], specifically for benefiting both their own health and that of their baby [72]. Women expressed how sharing data with HPs was “*reassuring*” and gave them a “*safety net*” [25] owing to a feeling of being monitored more closely by the HPs [73]. Similarly, some HPs believed that sharing data would provide an opportunity to review and monitor the data frequently [64,66], detect any changes or problems at an early stage [25,48] and thereby allow the early application of treatment or interventions for women with GDM [25,64,66].

Although some women and HPs felt comfortable communicating via technology [30,66,71,73,75], others were concerned about a lack of physical and emotional support [27,62,66,75] and a poorer quality of conversation [25,27,66]:

I like the one to one contact so you can ask questions. [25]

Nevertheless, women still felt there was a need to provide more interaction and communication between HPs and women via mobile app technology [76].

#### Theme 3: Usability of Technology

Women and HPs provided various perspectives on the usability of digital GDM systems in this theme. The content of the systems, including the quality of information and format and presentation of patients’ data, was the main usability aspect discussed by women and HPs in the included papers.

When women and HPs found the GDM systems “easy to use” [25,30,66,72,74], “simple” [25,66], “intuitive” [66] and “straightforward” [25,74], these impressions were influenced by the presence of simple language and images [63] and the simplicity of information presentation, such as displaying all data on one screen [62,72,77].

When usability concerns arose, they were also related to the data format and layout. Women and HPs suggested improving the layout and format of the information by changing the size of images or the amount of text [49], using videos [27], improving the data summary presentation [74,77], changing the data format to be similar to that of a paper logbook [25,48,63,74], and distinguishing different degrees of BG severity [48]:

To look back and see is there a blood sugar previous to try and identified yourself which was the pre and which was the post [meal test]. [25]

In the study by Pustozerov and Popova [76], HPs also indicated that improving the data format would help them review the data more easily.

Discussions on usability were also directed at the effectiveness of GDM apps in fulfilling the needs of women. Participants in different studies provided opinions about the lack of functionality in their GDM self-management systems. Some of their diverse suggestions included an option to scan barcodes of food [74], a time-alerting function for entering data [13], an educational or coaching feature [48], the ability to add a note to BG readings, and the ability to record the type of physical activity they have performed [65].

Women were also interested in having pop-up messages [65], informing them about any changes in their data [48], their condition [73], or any new activities in the forum [68] on the system:

To be able to review previous (entered) results and comments, to get an alert notice if results are out of the ideal range... [48]

A final aspect of usability concerned the effectiveness of information content. Women and HPs found the GDM information in both older technology [73] as well as that introduced by the studies [63,65,77] to be insufficient and simplistic [63,65,73,77]. Personalized information was considered vital [63] for diet [48,49] and in-depth information about GDM [65,77]. In addition, some women had issues with the clarity of the content and wanted simple, clear [49,77] and commonly used language [63,77] such as using “*tablespoon*” or “*bowl*” as familiar measurement units used by their dietitian rather than imperial measurements that were used to display food quantities on the app [63].

Despite the clear views of some women and HPs that using GDM self-management technology was more efficient in monitoring [48] and recording [68], other women were concerned about the inefficiency of their GDM systems [73,74]. Women with GDM found it was time consuming to use the system, particularly to retrieve information from food databases [74]. Postpartum women who had GDM perceived that the apps they used for GDM were overcomplicated and required too much commitment to complete a task [73]:

For something that was quite simple, it would take actually a long time to find it. [74]

I’ve never managed to do it for a long period, because of the amount of commitment. [65]

#### Theme 4: Discouragement Factors for the Use of Technology

The apparent disinterest of HPs was a cause of discouragement for women with GDM. Some said HPs lacked interest [63,65,71] and knowledge [65] in using technology. Indeed, their HPs’ preference for a paper logbook discouraged women from using digital GDM self-management tools [65], particularly those who were already unfamiliar with such technology [27]:

I had no interest in writing it two places, and I understood that no one was going to read or use my app…They always asked for my book, so I used that. [79]

Similarly, HPs were concerned about women’s abilities to use technology:

they’re all on their screens but at the end of the day, some of them don’t actually have credit to even look at a website or download a piece of information. [67]

Confirming this, some women reported little or no experience of using “message boards and things of that nature” [68]. Therefore, women themselves believed that some training might be needed to increase their confidence to use such technologies [27,72]. Some women with GDM were also concerned about the privacy of personal health information recorded on the systems [25,77].

In addition, HPs were concerned about the time required to use the systems and thought it would increase their workload [25,48,66,75]. They were also concerned that some women might not be able to afford the technology [75]:

We have some women who have got quite a low socioeconomic status, most of them still have phones...but not all have [mobile] data. [67]

Women with GDM and HPs also experienced technical problems as barriers to the use of GDM self-management technology. Both women and HPs reported some difficulties with data transmission [62,65,71], problems with accessing technology [75], and poor access to the local internet [25,30].

### Narrative Review of Quantitative Data

A narrative review was used to analyze the quantitative data, including the usability and user satisfaction results from 50% (13/26) of the included studies. Quantitative data from the remaining 27% (7/26) studies were not included in the analysis, as the results were not related to usability or user satisfaction [27,48,49,63,72,75] or were the result of objective task performance [77].

#### Usability

Quantitative studies used various measurements to gather data. Of these, only 12% (3/26) included a usability questionnaire to evaluate their systems, as summarized in Table 1.

Of the included studies, 8% (2/26) applied the system usability scale (SUS) developed by Brook in 1996 [85], with defined acceptability ranges for SUS scores (0-50 not acceptable, 50-70 marginal and 70-100 acceptable range) [86]. Jo and Park [13] reported a marginal score for their app, just below the acceptable threshold, (69.5 of 100). A similar, but acceptable, score was reported in the study by Gianfrancesco et al [74] for their web-based dietary system (70.9 out of 100) [74]. Pustozerov and Popova [76] included a custom questionnaire wherein women with GDM rated the “usefulness” and “convenience” of their GDM system on a 10-point scale. Usefulness was rated highly (8.7 out of 10), with convenience scoring somewhat lower (7.2 of 10).

In short, although these results suggest that previous GDM systems have usability challenges, it is impossible to draw any reliable conclusions with only 12% (3/26) studies providing results from a usability questionnaire.

**Table 1:** Included studies that used a usability questionnaire.

Usability	Type
Gianfrancesco et al [74]	SUS^a^ questionnaire
Jo and Park [13]	SUS (Korean version)
Pustozerov and Popova [76]	Custom usability questionnaire (10-point scale questions on *convenience* and *usefulness* + open-ended questions)

^a^SUS: system usability scale.

#### User Satisfaction

The included studies used different measurements to evaluate user satisfaction. Given et al [25] used an adapted version of the Telemedicine Satisfaction and Usefulness Questionnaire by Bakken et al [87]. Of the studies that included user satisfaction questionnaires, 4% (1/26) did not make their satisfaction questionnaire available [21], 12% (3/26) used specially developed satisfaction questionnaires [30,34,50], and the rest (4/26, 15%) used satisfaction questionnaires without any information on how they were developed [21,67,69-71]. Studies by Hirst et al [30] and Mackillop et al [43] were the only ones to provide evidence of the validity and reliability of their developed questionnaires.

The included studies reported generally high user satisfaction in their evaluations of GDM systems [21,25,30,34,50,67,69-71]. However, their user satisfaction questionnaires evaluated many different aspects of GDM systems regarding the type of technology and its features, making it difficult to clearly summarize areas for improvement. Table 2 shows the key measures of the user satisfaction questionnaires in the included studies (the complete measures are available in Multimedia Appendix 5). Most questionnaires used a Likert scale rating to assess the degree of participants’ agreement with their statements about the GDM systems. Women in these studies interacted with the technology within the period from GDM diagnosis until childbirth (usually between 8 and 10 weeks). They all used and evaluated the real working prototypes. Miremberg et al [21] were not included in Table 2 because the questions or satisfaction items were not available in their study.

Assessment of the aspects of convenience was common. Caballero-Ruiz et al [34] highlighted the convenience of minimizing travel to centralized clinics as the strongest indicator of satisfaction (approximately, on average, 9.5 out of 10). In other studies, women rated GDM apps highly for factors such as not complicating their lives [34,69,70] and the ability of these apps to fit into their lifestyles [30].

Improvement of GDM self-management was a highly rated aspect of the studied systems, including helping women to record BG levels [71], reminding them to take medication and record BG levels, helping them eat healthier, encouraging them to be more active [67], and helping to improve their GDM knowledge [34]. Moreover, most women found SMS text messages helpful and motivated them to optimize their GDM self-management [50,67]. A total of 2 studies also reported a general increase in women’s confidence in the management of their GDM [34,70].

Confidence or trust in GDM systems was rated well. Women with GDM reported confidence that the health care team checked their BG levels on the GDM system [71]. Many studies reported high ratings of confidence in the GDM systems, with women recommending them to others [34,43,50,67,69] or planning to use them in their next pregnancy [25,43,50,67]. Similarly, a study reported a high degree of trust (average 9 out of 10) in the GDM system [34], while another study reported that the GDM system was reliable [30].

Slightly lower satisfaction scores were reported for other aspects of ease of use: clarity of visualization of changes to treatment was rated approximately 7 out of 10 [34], and Peleg et al [70] reported satisfaction with system response time as approximately 3.5 out of 5, and ability to assist with interpreting self-monitored data approximately 3.8 out of 5.

Overall, based on the usability results (scores just under or above the acceptable threshold), there is much room for improvement in the usability of GDM self-management systems. However, with the limited number of papers providing a quantitative usability evaluation and the heterogeneity of questions assessing satisfaction, more studies are needed to identify where the improvement of usability and user satisfaction should be focused.

**Table 2:** User satisfaction question topics in the included studies.

Summary of key measures of user satisfaction questionnaires	Study								
	Varnfield et al [71]	Johnson and Berry [67]	Mackillop et al [43]	Peleg et al [70]	Caballero-Ruiz et al [34]	Peleg et al [69]	Bartholomew et al [50]	Hirst et al [30]	Given et al [25]
Convenient			✓^a^					✓	
Avoiding displacement					✓				
Fit in with life or did not complicate it			✓	✓	✓	✓		✓	
Adapt to daily life and context changes				✓		✓			
Number of hospital consultations is enough			✓		✓				
Help to record BGLs^b^	✓								
Help to remember to take medication and take BG^c^		✓							
Help to eat healthier or become more active		✓							
Helps to improve GDM^d^ knowledge					✓				
Increased motivation for self-management							✓		
Improved diabetes control							✓		
Help to feel confident in managing GDM	✓			✓		✓			
Feel confident that health care team checked BGLs	✓								
Recommending to others		✓	✓	✓	✓	✓	✓		
Using it again		✓	✓	✓		✓			✓
Useful				✓	✓				
Easy to use				✓			✓		✓
Ease to learn how to use				✓	✓				
Helps data interpretation				✓	✓				
Clarity or effectiveness of visualization				✓	✓				
Clarity of activities’ sequence in app				✓					
Personalized							✓		
System response time				✓		✓			
Experiencing error with the system				✓					
Time consuming							✓		
Trust is being well controlled					✓				
Trust it to work									✓
Reliable to use			✓					✓	
Satisfaction regarding diabetes follow-up					✓				
Satisfied with the system	✓								✓
Enjoyable or interesting				✓			✓		
Paying for the system				✓		✓			

^a^✓: illustrates where a study included a measure of user satisfaction in its participant questionnaire.

^b^BGL: blood glucose level.

^c^BG: blood glucose.

^d^GDM: gestational diabetes mellitus.

## Discussion

### Principal Findings

#### Overview

The primary objective of this systematic literature review was to identify the views of HPs, women with GDM, and postpartum women who have had GDM regarding GDM self-management technology. The secondary objective was to investigate the usability and user satisfaction levels of existing technologies and quantitatively evaluate these factors.

Regarding the first objective, thematic analysis of the qualitative data in the selected papers identified four themes: (1) the benefits of using technology, (2) engagement with people via technology, (3) usability of technology, and (4) discouragement factors for the use of technology.

The thematic analysis of qualitative data revealed barriers to usability, including technical problems, data privacy, lack of emotional support, the accuracy of reported data, and adoption of the system by HPs. Convenience, improving GDM self-management, peer support, increasing motivation, increasing independency, and providing consistent monitoring were common facilitators of using this technology.

For the second objective, the narrative review of the quantitative data (usability and user satisfaction) showed that there is room for improvement in the usability of GDM self-management systems.

#### Benefits of Using Technology

##### Convenience of Technology Use

The influence of convenience in our analysis, in both the qualitative and quantitative findings, is echoed in other literature on telemedicine. Pérez-Ferre et al [88] reported a 65% reduction in the number of clinical visits for women with GDM who were using telemedicine. The main benefits of doing so are the improvement of HPs' work efficiency and a better quality of life for women with GDM [57].

Although our findings indicated a strong positive desire to reduce in-person clinics through technology, not everyone wanted clinical visits replaced altogether. This was affirmed in a recent systematic review that highlighted the negative impact of losing in-person contact between women with GDM and HPs [57], particularly for women who experience social isolation and anxiety during pregnancy [89]. However, these studies were carried out before the COVID-19 pandemic. Today, patients may be more familiar with remote consultations, and the impact of this would benefit from further investigation.

##### Improving GDM Self-management by Using Technology

Our results revealed that women appreciated the use of technology to manage various aspects of their condition. These findings are consistent with those of relevant studies outside the scope of this review. Leziak et al [90] explored the experiences of women with GDM and pregnant women with type 2 diabetes using mobile health (mHealth) during pregnancy. Their results showed enhanced self-management through the use of mHealth technology [90]. Similarly, Yee et al [91] explored how pregnant women with GDM or preexisting diabetes perceived an SMS-based intervention during their pregnancy, showing an optimization of GDM self-management and increased motivation for diabetes self-care. In 2007, Homok et al [32] evaluated the feasibility of a web-based telemedicine system that monitored the BG levels of underserved (poor socioeconomic status) women with GDM using the Diabetes Empowerment Scale [92]. Participants experienced increased diabetes management self-efficacy, such as readiness to change their lifestyle behaviors to achieve diabetes goals.

In summary, evidence suggests that technology could help women optimize their GDM self-management abilities, leading to benefits for both themselves and their baby’s health. As a result of good practices initiated through GDM self-management technology, women could also improve control over their health, which could be maintained habitually after giving birth to prevent the development of type 2 diabetes.

#### Engagement With People via Technology

As mentioned earlier, this theme consists of 2 main components: “engagement with peers” and “engagement with health care professionals.”

The results of the thematic analysis demonstrated the benefits of peer support in digital GDM self-management systems [68,73,75] a finding supported by similar studies outside the scope of this review. Leziak et al [90] explored the experiences of low-income women with GDM and pregnant women with type 2 diabetes, using mHealth technology to support and improve diabetes self-management during pregnancy. Their results highlighted how women valued social interactions with other women and accessed their knowledge and experiences. McMillan et al [93] evaluated mHealth technology to support postpartum women with a history of GDM in maintaining postnatal activity and good dietary habits, finding that a discussion forum was a valuable feature in doing so [93]. As other previous studies have emphasized, such favorable opinions of women toward peer support stem from their ability to share or read stories about other women [91] and receive emotional support [94], which is an important factor in health communication [95,96]. Indeed, some HPs believed that pregnant women valued other women’s experiences more than HPs’ advice during their pregnancy [97]. However, Sherman and Greenfield [94] found that, when examining message boards for pregnant teenagers, some of the medical information posted by pregnant women was misleading because it was suitable for their specific condition and therefore inappropriate for others [94]. Furthermore, validation of posted information is also a major challenge [95], and further work is needed in this area to provide a reliable and validated communication path between women with GDM.

Our thematic analysis described women’s interest in sharing data with their clinicians by remote means, to obtain reassurance and to be monitored more consistently. This is also evident in some previous studies. Dalfra et al [31] found that women with GDM and pregnant women with type 1 diabetes appreciated their telemedicine system for sharing their data with HPs and their ability to communicate with them whenever needed. Similarly, Leziak et al [90] showed that women were also in favor of sharing data with HPs and receiving real-time feedback. However, in the included studies, some HPs found it difficult to trust women’s reported data [72,76]. In contrast, Kruger et al [98] found that HPs were satisfied with the accuracy of the data reported by women with GDM via a telemedicine system. Other studies have found that it is unlikely that women would misreport their records, as they are highly motivated to maintain BG control [31] for the sake of their baby’s health [57]. Further work is needed to examine the means of decreasing the possibility of reporting incorrect data.

#### Usability of Technology

Although the evidence available regarding the usability of digital GDM self-management systems is limited [99], the findings of our review are in line with those of previous studies on mHealth self-management systems for type 1 and type 2 diabetes. Katz et al [100] assessed 8 current diabetes self-management apps for adults with type 1 diabetes, discovering issues in the interpretability of data and high cognitive load. These results were corroborated by Fu et al [101] in an evaluation of 4 apps for type 2 diabetes management. Further studies have also found usability challenges with data format on mHealth self-management systems [102-104], such as difficulty interpreting or understanding data in its current format [104]. A useful digital self-management system should display data trends and patterns, specifically showing which data are normal or abnormal. Usability issues with data formats thus prevent patients from understanding their data [105,106], thereby limiting their self-management capabilities.

Our review also identified limitations in the functionality of the systems as another usability concern across the included studies. Previous reviews of general diabetes self-management apps have highlighted important missing functionality, including automatic transfer of BG data from a glucometer to a mobile app, personalized diabetes management advice [107], prevention of errors [108], freedom to edit or remove data entries and appointments, and the ability to automate common tasks [109].

The limited functionality of diabetes self-management systems can be considered a usability problem [109] and is likely to result in these systems failing to meet users’ needs [107]. Addressing these functionality limitations would mitigate some of the usability challenges and help users optimize their engagement and interaction with these systems.

Quantitative evaluation of GDM self-management apps in the studies by Jo and Park [13] and Gianfrancesco et al [74] yielded SUS scores just below and above the acceptable threshold, respectively. Unsurprisingly, previous studies that used the SUS questionnaire to evaluate diabetes self-management apps in different domains have received similarly poor ratings [101,110,111]. Similar to this systematic review, these previous studies used guidance from Bangor et al [85] to interpret the SUS scores, with most apps falling below the acceptable range.

Our quantitative analysis identified the need to improve the usability of GDM self-management systems. However, with the limited number of papers providing a quantitative usability evaluation, the heterogeneity of questions assessing satisfaction and the variation in systems being assessed, it is difficult for quantitative studies to identify where the improvement of usability and user satisfaction should be focused. Therefore, it is an aspect that needs further investigation.

#### Discouragement Factors for the Use of Technology

Despite the perceived benefits of GDM technology, our analysis revealed technical problems as a prevalent barrier across the included studies. Previous studies have reported similar technical problems when using eHealth and self-management systems [102,112-115]. Moreover, a previous systematic literature review by Simblett et al [116] identified technical problems as one of the most significant barriers to using mHealth technologies. The most common technical problems in their review were app disappearance, loss of power, restarting without warning, not receiving notifications, receiving them at the wrong time, and having a difficult connection. Indeed, 2 participants withdrew from one of the included studies because of difficulties with internet connectivity. Parallel to the findings of this review, technical problems were the cause of reducing participants’ motivations [112,113] and even the cause of leaving the study by participants with other health conditions [114,116].

In addition to technical problems, the privacy of personal health information was a concern for some women. Simblett et al [116] also reported privacy concerns in one of the included studies. Although the use of advanced encryption algorithms and pseudoanonymization of personal data should address security and privacy challenges at the system level, it is important for future GDM systems to effectively communicate good security practices to reassure new users [117].

Although most women across all studies were interested in using self-management technology, some suggested that their HPs were disinterested. Similarly, Wake et al [118] recognized the lack of awareness and adoption of technology by HPs as an important barrier to using eHealth for diabetes self-management [118]. HPs’ difficulty to accept technology was experienced in previous studies [119-121], influenced by difficulty integrating it with their workflow [102,121], lack of integration with the medical record system [120], or a lack of technical knowledge [116]. Further work is required to involve HPs in the design and development of GDM technology more effectively to reduce this barrier.

### Limitations and Further Work

The strengths of this review were its application of a rigorous process in paper selection and summarizing results that include both qualitative and quantitative data to cover a wide scope of understanding. Although this systematic literature review was conducted by the first author, we mitigated the potential for bias through a double screening of a proportion of papers’ citations (title and abstract) by the entire research team, in line with previous systematic literature reviews published in JMIR. Two of the authors were also involved in theme development and the methods and results were reviewed by all authors.

Thematic analysis was restricted to the qualitative data contained in the papers (19/26, 73%). It is possible that the authors of the included studies did not report significant results. However, it is unlikely that the key findings were not reported in the original papers.

The details of the methods and methodologies applied were limited in some studies. The available evidence is also limited by several factors. First, some studies used small sample sizes. Methodologically robust trials of greater sizes are needed to confirm the findings of our review. Second, the number of quantitative studies that measured usability was limited. Third, most of the evaluations of satisfaction did not address the validity and reliability of the satisfaction questionnaires. Furthermore, some questions in the satisfaction questionnaires were generic. Using standard evaluation tools and valid questionnaires would offer consistent and robust results across different studies.

Overall, further work is required to improve the usability of GDM self-management systems. There is a need to evaluate the systems using various usability approaches [109,122,123] and larger samples to obtain broader usability perceptions and identify problems with the systems. Furthermore, more engaging elements in a GDM self-management system are needed to develop better emotional support for women. Work is needed to improve peer communication to develop more support for women with GDM.

Further work is also needed to assess the design and development process of these GDM self-management technologies that might help identify the source of these usability challenges.

### Conclusions

This is the first systematic literature review to carry out a comprehensive review of the perspectives of HPs, women with GDM, and postpartum women who have had GDM about using technology for GDM self-management during pregnancy. Despite the existence of several studies on technology and GDM, information about the perceptions of women with GDM and HPs regarding GDM self-management technology is limited. More rigorous studies are needed to reveal evidence-based barriers to and facilitators of using existing GDM self-management systems.

## Appendix

Multimedia Appendix 1 Keywords and search strategy.

Multimedia Appendix 2 Study characteristics.

Multimedia Appendix 3 Quality assessment.

Multimedia Appendix 4 Definition of the themes and subthemes.

Multimedia Appendix 5 Satisfaction measurements.

## Abbreviations

BG: blood glucose
GDM: gestational diabetes mellitus
HP: health professional
mHealth: mobile health
PRISMA: Preferred Reporting Items for Systematic Reviews and Meta-Analyses
RCT: randomized controlled trial
SUS: system usability scale
