# Supplementary material for: Barriers to and Facilitators of Using eHealth to Support Gestational Diabetes Mellitus Self-management: Systematic Literature Review of Perceptions of Health Care Professionals and Women With Gestational Diabetes Mellitus
Source: J Med Internet Res. 2022 Oct 27;24(10):e39689. doi: 10.2196/39689 (PMC9650580; doi:10.2196/39689)
Supplement: Multimedia Appendix 1 [file jmir_v24i10e39689_app1.docx]

Multimedia Appendix 1

Medline, Cinahl and Embase

Keywords for Self-management set

1. Self-management, 2. Self-monitoring, 3. Self-control, 4. Self-care, 5. Patient centrality, 6. User center, 7. User centre, 8. User centred design, 9. Patient control, 10. Patient directed care, 11. Patient-led, 12. Patient-centered, 13. Patient-centred, 14. Patient manage*, 15. Manage*, 16. Monitor*, 17. Control*, 18. Self directed exercises, 19. Patient compliance, 20. MH “Self Care”, 21. MH “Prenatal Care*”, 22. MH “blood Glucose Self-Monitoring” (Medline and Cinahl) or “blood Glucose Self-Management” (Embase).

23. 1 or 2 or 3 or 4 or 5 or 6 or 7 or 8 or 9 or 10 or 11 or 12 or 13 or 14 or 15 or 16 or 17 or 18 or 19 or 20 or 21 or 22

Keywords for Gestational diabetes set

24. Gestational diabetes mellitus, 25. GDM, 26. Pregnan* diabetes, 27. Pregnan* DM, 28. Pregnan* diabetes mellitus, 29. Women DM, 30. Pregnancy in Diabetic*, 31. Gestational hyperglycemia, 32. Hyperglycemia in pregnancy, 33. Diabetes in pregnancy, 34. Pregnancy in diabetes, 35. Diabetic pregnancy, 36. MH “Diabetes, Gestational” (Medline), MH “Diabetes Mellitus, Gestational” (Cinahl) or MH “Pregnancy diabetes mellitus” (Embase).

37. 24 or 25 or 26 or 27 or 28 or 29 or 30 or 31 or 32 or 33 or 34 or 35 or 36

Keywords for Technology set

38. Telemedicine, 39. Pervasive healthcare, 40. Ehealth, 41. e-health, 42. ehealth, 43. mhealth, 44. m-health, 45. Electronic personal health record, 46. Decision support system, 47. electronic records, 48. electronic health, 49. mobile health, 50. smartphone technology, 51. patient guidance system, 52. internet*, 53. web-based, 54. web*, 55. wireless, 56. SMS, 57. Short messag*, 58. digital*, 59. digital health, 60. tele*, 61. technology, 62. comput*, 63. Intervention, 64. *phone*, 65. Video*, 66. Mobile*, 67. Remote*, 68. PDA, 69. Smartwatch app*, 70. Smartphone, 71. MH “Telemedicine”, 72. MH “Electronic Health Records”, 73. MH “Computer Communication Networks”, 74. MH “Mobile Applications”, 75. MH “Smartphone”, 76. MH “Mobile Phone” (Embase) or “Cell Phone” (Medline) or “Cellular Phone” (Cinahl), 77. MH “Text Messaging”, 78. MH “Technology”, 79. MH ”Internet”, 80. MH “Decision Support System, Clinical”.

81. 38 or 39 or 40 or 41 or 42 or 43 or 44 or 45 or 46 or 47 or 48 or 49 or 50 or 51 or 52 or 53 or 54 or 55 or 56 or 57 or 58 or 59 or 60 or 61 or 62 or 63 or 64 or 65 or 66 or 67 or 68 or 69 or 70 or 71 or 72 or 73 or 74 or 75 or 76 or 77 or 78 or 79 or 80

82. 23 and 37 and 81

IEEE

Gestational diabetes mellitus or GDM

ACM

Gestational diabetes mellitus or GDM

Notice: As above shown, some of the keywords were adjusted in order to match the requirements of each database.
