# Supplementary material for: Barriers to and Facilitators of Using eHealth to Support Gestational Diabetes Mellitus Self-management: Systematic Literature Review of Perceptions of Health Care Professionals and Women With Gestational Diabetes Mellitus
Source: J Med Internet Res. 2022 Oct 27;24(10):e39689. doi: 10.2196/39689 (PMC9650580; doi:10.2196/39689)
Supplement: Multimedia Appendix 2 [file jmir_v24i10e39689_app2.docx]

Multimedia Appendix 2: Study characteristic

N/A. Not Applicable N/S. Not Stated

Part 1

| First Author | Design | Location | Number/ Age of  participants | Inclusion/ Exclusion criteria | Duration of study or participants’ intervention/ Type of intervention/ Name of technology | Analysis method |
| --- | --- | --- | --- | --- | --- | --- |
| Bartholomew [50] | Quantitative study based on RCT. | The Kapi’olani Medical Centre, America | Number: 100  Age: Average age 33.2 ± 5.37 years | Inclusion: Age ≥ 18, women with GDM or type 2 who were before 30 weeks and one day of gestation.  Exclusion: N/S | Study duration:  Roughly 12 months recruitment, 6 weeks duration for involvement of participants in the study  /Cell-phone internet technology/ N/S | Student’s t tests and also χ2 test for categorical data |
| Bromuri [64] | The architecture of a system, and Mixed methods based on RCT and qualitative questionnaire (participant’s perception) | University Hospital of Lausanne  (Sweetzerland) | Number: 24,  Age: Average 32 ±5 | Inclusion: Speaking French, having GDM within *>*24^th^  and *<*32^nd^ weeks of pregnancy,  Exclusion: N/S | Study duration: From February 2013 to June 2013/ Personal Health  System integrated with a galaxy smartphone to monitor  GDM/ N/S | Univariate quantitative  analysis for quantitative data.  The type of qualitative analysis method has not been specified. |
| Caballero-Ruiz [34] | Quantitative study based on RCT and patients’ satisfaction. | Parc Tauli University Hospital,  Spain | Number: 90 patients (60 intervention group and 30 control group).  Age: N/S | Inclusion: Women with GDM  Exclusion: N/S | Study duration: 17 months / Web-based telemedicine care / Sinedie | Descriptive statistic was used for clinical outcome and 10 point semantic deferential scale for patient satisfaction of the system. |
| Carolan-Olah [41] | A mixed-methods based on Pre-test/post-test surveys | Australia | Number: 21 women with GDM  Age: Most of women were under 35 years. 16 of participants were under 35 years, 5 participants between 36-45 and one not known | Inclusion: Pregnant women with GDM, age >18 years, singleton pregnancy and English speaking.  Exclusion: N/S | Participants: Interacting with website while waiting for their clinical appointment. 5 min time for each of the pre and post questionnaire to complete./ GDM information website that can be accessible from computer, tablet or smartphone/ N/S | Fisher’s exact test for finding improvement in each knowledge scale. |
| Edwards [73] | Qualitative study with semi-structured interviews. | UK | Number:10  Age: N/S | Inclusion: Having GDM within the past five years  Exclusion: N/S | Interview duration 30-45 min /mHealth experience / N/A | Braun and Clarke’s Thematic Analysis method. |
| Garnweidner-Holme [62] | Qualitative study based on interviews. | Oslo, Norway; 5 diabetes  outpatient clinics | Number: 9  Age: N/S | Inclusion: HPs who provided care to pregnant women with GDM who were in the Pregnant+ RCT study.  Exclusion: N/S | Qualitative data (interview) collected between May and June 2017, duration of interviews were approximately 16–35 min. /Mobile Application/ Pregnant+ | Thematic analysis (Braun and Clark’s method) |
| Garnweidner-Holme [77] | Development and Usability (a mixed method based on interviews and Task Performance). | Norway | Number: 22  Age: N/S | Inclusion: Diagnosed with GDM, no medicine needed for treatment of diabetes, and also had an experience in recording blood glucose  Exclusion: N/S | N/S /Mobile Application / Pregnant+ | Quotation count report for analysing the interviews |
| Gianfrancesco [74] | A mixed- methods with prospective observational study based on measuring submission of food records on the system, the SUS questionnaire and interviews. | Leeds Teaching Hospital NHS Trust Diabetes, UK | Number: 199  Age: Average 33.3 years (SD 5.0) | Inclusion: Women diagnosis of GDM who attend their first clinical visit after their diagnosis, ability to read and understand English, They have not started any diabetes medication.  Exclusion: N/S | Participants: Two weeks period/ Online Dietary tool/ myfood24 | Descriptive statistic, independent t-test for continuous data, chi squared test for categorical data, and thematic analysis (Braun and Clark) for qualitative data. |
| Given [25] | A mixed-methods based on RCT, semi structured interview and patient’s satisfaction questionnaire. | Northern Ierland | Number: 50  Age: Average age for control group= 30.1± 5.5 (26) and for telemedicine group= 33.5±4.2 (24) | Inclusion: Ability to use the telemedicine devices, adequate communication ability, willing to use glucometer during the study.  Exclusion: Pre-existing diabetes type 1 or 2, consuming oral steroid therapy | Participants: Roughly 10-12 weeks /Telemedicine hub / N/S | Descriptive statistic and framework analysis |
| Harrison [27] | A mixed-methods Pilot study based on survey and interviews | Kaiser Permanente Southern California | Number: Phase 1: Survey =70 women,  Phase 2: interview =10  Phase 3: N/S  Age: Survey: 25 women were between 18 and 29 years, 42 women between 30 and 39 years, 4 women were 40 or above.  The average age of women for the interview was 31.5 years. | Phase 1: N/S, Inclusion for Phase 2 interview: Between 18 and 45, less than 32 weeks gestation at the time of registration, have a plan to attend local facility for delivery, a diagnosis of GDM.  Exclusion for Phase 2: Multiple pregnancy, having history of a fetal dead after 10 weeks, chronic health condition, for more than 2 weeks consumed oral steroid, having a history of bariatric surgery, if their smartphone does not have internet connection. | Study duration: 2014-2015 /Telemedicine / N/S | Analytic induction (Bradley, Curry, & Devers, 2007). |
| Hirst [30] | A mixed-methods pilot study  Based on patient’s satisfaction questionnaire and acceptability of the developed app. | Oxford University Hospitals NHS Trust, Oxford, UK. | Number: 52  Age: mean age was 34.8 years with SD 5.232 for women with new diagnoses of GDM, and for the remaining 17 women with previous GDM mean age was 30.4 years with SD 4.3 | Inclusion: Diagnosed with GDM before 34 weeks of gestation, no need for pharmacological therapy after monitoring BG for a week, with singleton, uncomplicated pregnancies with the ability to understand.  Exclusion: N/S | From diagnosis with GDM until childbirth / Mobile Application / GDm-Health | Factor analysis |
| Jo [13] | Development and a mixed methods based on usability and acceptancy. | Seoul, Korea | Number: 5 GDM women participated for usability evaluation.  36 GDM women participated for testing the acceptancy of the system.  Age: Mean age of participants (acceptancy test) = 32.2 years | Inclusion for testing of User Acceptance: being pregnant, having a diagnosis of GDM, and owning an Android smartphone.  Exclusion: N/S | Participants: 1 week / Android smartphone  Application / N/s | Descriptive statistic |
| Johnson [67] | Quantitative study based on feasibility and user satisfaction | University of North  Carolina | Number: 19  Age: 31.1 ± 5.6 | Inclusion: Over 18 years, diagnosed GDM by two or more Glaucous tolerance test, owing mobile phone and ability to read and write in English.  Exclusion: N/S | Participants:  4 weeks /Text messaging /  N/S | Descriptive statistic |
| Khalil [66] | Qualitative study based on semi-structured interviews. | 12 different health care  centers in France. | Number: 20 HPs and 15 patients. | Inclusion for HPs: working in diabetes services in France and having experience in both telemonitoring and traditional follow-ups.  Inclusion for women: diagnosed with GDM and during at least 1 pregnancy have used glucometer and telemedicine (myDiabby). | Interview duration average 30 minutes /Telemonitoring (application) / myDiabby app | Thematic analysis approach. |
| Mackillop [43] | Mixed methods study based on randomized controlled trail and satisfaction questionnaire. | Oxford University hospital, UK. | Number: 206 (Intervention group: 103,  Control group: 103) | Inclusion: Women with GDM, between 18 and 45 years with a viable singleton pregnancy and less than 35 weeks | Participant Median time: 54 days / Mobile application /  GDm-Health | Linear regression  Equation, nonparametric test, logistic regression, descriptive statistics |
| Miremberg [21] | Quantitative study based on Randomized  controlled trial. | university-affiliated medical  Centre. No more information available. | Number: 126  Age: 31.7 ± 4.2 years for smartphone group,  and 32 ± 6.3 for control group | Inclusion: 18-45 years, singleton gestations, first diabetes-in pregnancy clinic visit <34 gestational weeks and enable to understand English. | Study duration: 12 months from May 2016 to May 2017 /Mobile Application / N/S | X2 test or Fisher  exact test and Student  t test |
| Nicholson [68] | Mixed methods Pilot study based on  interview, feasibility and Post-intervention focus group. | The University of North Carolina, US. | Number: Phase 1=10,  Phase 2=23  Age: Phase 1: average age was 33 (23-44).  Phase 2: 31.7 (sd=4.7) | Inclusion:  Phase 1: Women with GDM, and English speaking.  Phase 2: women 21 years or older, recently diagnosed by GDM, English speaking, having computer, and also internet access.  Exclusion: N/S | Participants: From within 2  weeks of GDM diagnosis until  36 weeks of pregnancy/ Website/  GooDMomS | Phase 1: grounded theory  Phase 2: descriptive analysis, chi-square statistic, sensitivity analysis, paired t-test. |
| Pais [72] | Development and a qualitative study with a user-centered design approach based on interview. | Auckland, New Zealand | Number: 5 clinicians contributed initially, then 10 including 5 other clinicians and 5 GDM patients participated to evaluate the ecosystem prototype  Age: N/S | Inclusion for participants: N/S  Inclusion for  Mobile apps: Apps with food diary and physical activity functions which allowing data sharing with third party and also to be free to download.  Exclusion: N/S | Study duration: almost one year / Reviewing mobiles app properties and functionalities./ Ecosystem of mobile apps | Analysed qualitatively |
| Peleg [69] | Quantitative study based on feasibility using Quasi-experimental . | Spain | Number: 19  Age: 35.2 ± 3.9 | Inclusion: Women with GDM  Exclusion: N/S | Participants: Average 57.1 days / Mobile Application / MobiGuide | Descriptive statistic was used for clinical outcome and 5 point semantic deferential scale for patient satisfaction. |
| Peleg [70] | System architecture and mixed methods based on the knowledge elicitation and specification methods and feasibility study | Spain for GDM | Number of women with GDM: 20  Average women with GDM age: 35 | N/S | GDM: between 2-5 months/ Mobile application/ MobiGuide | The Wilcoxon signed rank test, descriptive statistic and 5 point semantic deferential scale for patient satisfaction. |
| Pustozerov [76] | Mixed methods study based on pilot post- study Usability. | Saint Petersburg, Russia | Number: 33 women and 4 endocrinologists  Age: N/S | N/S | Participants: During pregnancy period / Mobile and Desktop Application/ DiaCompanion; application | N/S |
| Rasekaba [75] | A mixed-methods study based on semi-structured interviews and clinical information. | Victoria,  Australia. | Number: 9 women with GDM and 3 clinician and 2 IT staff.  Age: 7 women between 25 and 34,  2 women≥ 35 | Inclusion: Pregnant women diagnosed with GDM, age 18 or over,  IT staff from the health service’s IT department.  Exclusion: N/S | Clinical data collected for 12 months (October 2016- October 2017), Duration of interview 15-45 minutes/ TeleGDM web-based Telemedicine technology. | Descriptive analysis used for quantitative data and thematic analysis for qualitative data |
| Skar [65] | Qualitative study based on semi-structured  Interviews using Interpretative  phenomenological method. | Norway | Number: 17  Age: N/S | Inclusion: Women from intervention group from 5 diabetes outpatient clinics from previous study.  Exclusion: N/S | Qualitative data (interview) collected between October 2016 and February 2017, duration of interviews were 30 min / Mobile Application/ Pregnant+ | Interpretative phenomenological analysis method (IPA). |
| Surendran [63] | A mixed-methods study based on interviews, frequency of application usage and health information. | National University Hospital, Singapore | Number: 340 women with GDM for RCT,  14 women for interview.  Age: mean 32 | N/S | Quantitative data collected for 2 months (June and July 2019), qualitative data gathered during 3 months (interview, November 2019- January 2020 ), duration of interviews not available /Mobile GDM application /Habits-GDM | Descriptive statistics used for quantitative data and Braun and Clarke thematic analysis method for qualitative data. |
| Varnfield [71] | Development and a mixed methods study based on feasibility, user adaption and user satisfaction (Likert scale and open ended questionnaire). | Redland Hospital, Queensland, Australia | Number:40  Age: between 23 and 42 years | Inclusion: diagnosis of GDM between 24 and 28 of gestational weeks with a confirmed oral glucose tolerance test, at least 16 years, owning a smartphone and the ability to use it, and speak and understand English. Exclusion: women with any other diabetes types, severe comorbidities that would limit participation, history of major psychiatric illness. | Participants: From diagnosis with GDM to childbirth (roughly between 11-16 weeks) / mobile and desktop application for women’s side and  data collection and processing software for the centralized  server / M♡THer app | Descriptive statistic, questionnaires (including open ended questions) were analysed in description form. |
| Wickramasingh [48] | A mixed-methods pilot study based on  quasi-experimental design with using a two-period crossover strategy. | A private hospital in Melbourne,  Australia | Number: 10 patients, two obstetrics and three diabetes educators  Age: N/S | Inclusion: Diagnosed with GDM between 26 and 28 weeks of gestation by glucose tolerance test.  Exclusion: N/S | Participants: 8-week period/ Mobile Application / DiaMonD (diabetes monitoring  device) | Thematic analysis for qualitative data; simple regression techniques and exploratory data mining techniques for quantitative data. |

Study characteristic

N/A. Not Applicable N/S. Not Stated

Part 2

| First Author | Study goal | Quantitative /Qualitative data collection tool | Key findings related to the systematic review objectives |
| --- | --- | --- | --- |
| Bartholomew [50] | To compare a cell phone–Internet technology (CIT) with a conventional voicemail system for managing women with GDM. | Compliance with SMBG reporting and Patient satisfaction. / N/A | The results showed that satisfaction scores for the intervention group (using CIT) were significantly higher than the control group (voicemail system). |
| Bromuri [64] | To present the architecture of Personal Health  System, and assess its  feasibility and women’s and clinicians’ acceptability of the system. | Clinical outcomes, demographic data and part of the questionnaire/ Questionnaire to gather perceptions of women in a telemedicine group and HPs, a focus group only with HPs | The majority of women in the telemedicine group were very satisfied with the care that they received. All participants found the data entry and smartphone system were easy to use.  Healthcare professionals believed that telemedicine technology was an appropriate way to manage GDM conditions. In addition, it helped to save time, improved the quality of care, and increased to react quicker to any changes in women’s data. |
| Caballero-Ruiz [34] | To compare Sindie web-based system and standard care in order to understand the effectiveness and safety of Sindie system. | Satisfaction questionnaire / N/A | Patients rated the following satisfaction questions highest (between 9-10 from 10):  “Using the system avoid displacement”, “Number of hospital consultation is enough” and “Trust in being well controlled”.  Patients rated the following satisfaction questions Lowest (between 7-8 from 10)  “Clarity of visualization treatment changes” and “No feel pressure about sending data frequently”. |
| Carolan-Olah [41] | To develop and evaluate a GDM information website. | Questionnaire / 3 free text questions to explore patients’ opinions. | 70.6% of participants improved their GDM knowledge score after interacting with the website. 50% of the participants improved their food knowledge score and 38% of the participants improved their self-management score after interacting with the system. 85.7% of the participants found the system useful and easy to use. In general, the website helped women with lower education to improve their knowledge about GDM, food and self-management compared to women with higher education. |
| Edwards [73] | To explore experience and perceptions of women who have had GDM during last 5 years about using mHealth technology during and after pregnancy. | N/A / Semi-structured interview | Main findings for using mHealth: Reduce traveling, improve self-management ability, increase reassurance of being monitored, be pervasive of tech, and usefulness of Facebook Forum for GDM self-management. All women trust the information provided by other women on Facebook Forum.  Main findings of the current care: Access to inadequate GDM information and Delay to access to information |
| Garnweidner-Holme [62] | To explore healthcare professionals’ perceptions about provision of care to women with GDM who used Pregnant+ app to manage their GDM condition in a randomized controlled trial. | N/A / Interview with HPs about the Pregnant+ app. | HPs’ opinions about the advantages of using mHealth for women: Accessing trustworthy information all the time, the convenience of recording blood glucose compared to a logbook which can be easily lost and the cultural sensitivity of the app.  Healthcare professionals reported technical problems emphasizing the automatic transfer of blood glucose reading as a significant barrier for using the mobile app. |
| Garnweidner-Holme [77] | To document the design and development procedure of Pregnant+ app. Also to conduct user-involvement studies to assess Pregnant+ app prototype. | Task performance (%) / Interview women with GDM. | Suggestions for improving the first pregnant+ prototype: Using easier language, including more information about the risk of GDM for both mother and child, using a larger picture, and providing emoji feedback on different blood glucose levels in a graph.  Benefits: easier to use a mobile phone compare to a booklet due to being with you all the time. There is a potential to miss recording on the booklet and has to look for a pen.  Concern: Privacy of the personal health information recorded in the app was a concern by some of the users. |
| Gianfrancesco [74] | To assess the feasibility and usability of an online food recording called myfood24 system for women with GDM. | SUS questionnaire / Interview | The SUS was reported just above the acceptable threshold (70.9 out of 100).  Women found myfood24 straightforward and easy to use. All women found the system useful in providing instant food neutrinos summary. Women reported using the system was more accurate but was less convenient than paper diaries due to its format.  Suggestion:  Women: Improving accessibility of the system by developing an app to be convenient and accessible via mobile phone during the day. Improving the usability of the system by having the option to scan bar codes and an easier way to retrieve their favorite foods.  HPs: there is a need for a function to record the blood glucose reading alongside food tracking. Improving visual displays of the nutritional summary. Ability to track the data and personal target. |
| Given [25] | To assess the feasibility and acceptability of using telemedicine for GDM management and replacing a few of Face-to-Face consultations with using telemedicine. | Satisfaction questionnaire / Interview women and HPs | Majority of women (89.4%) in the telemedicine group and HPs agreed or strongly agreed on being satisfied with the telemedicine system and using the system again. Women reported telemedicine devices easy to use.  Technical problem: Having difficulty using the website. Having problems with transferring data (took too long) and not being always confident about telemedicine devices working.  Women’s concerns: clinical visits not to be replaced completely by telemedicine and privacy concerns.  Healthcare professionals’ concerns:  Difficult to interpret the long list of data.  Being a bit slow (due to the system itself or internet connection), missing direct communication, and analysing and reviewing telemedicine data would create additional pressure on health care professionals. However, Some of the HPs believed that telemedicine would decrease the clinician workload. |
| Harrison [27] | To assess acceptability of a telemedicine system that transfer women’s BG, BP and weight information to HPs. | Survey / Interview and open ended questions on the survey. | Participants had a positive conception of the proposed system due to having less absence from work, reduced traveling and waiting time to attend the clinical visit, and a decrease in the need of childcare.  Most of the participants mentioned that they would be comfortable using the proposed telemedicine with virtual visits. One patient mentioned that because of IVF her pregnancy is a high-risk pregnancy and she preferred face-to-face visits to virtual visits through the phone. |
| Hirst [30] | To assess women’s satisfaction about using GDm-Health app to manage their GDM condition and also satisfaction about their attitudes toward their diabetes care. | Satisfaction questionnaire / Feedback at the end of the questionnaire. | Majority of women ranked satisfaction measures for the GDm-health system agreed or strongly agreed.  It was reported that the phone was easy to use. Women preferred the use of technology than to travel and wait at the clinic, especially if they had other children.  Some of the patients commented that they had a problem with a poor local network (3G) So they couldn’t transfer the data automatically. One of the participants left the study because of a poor network. |
| Jo [13] | To document the development and to evaluate the GDM android smartphone app. | Korean version of SUS questionnaire and acceptancy questionnaire /Participants feedback | Usability scores were from 52.5 to 87.5. The average usability was 69.5 of 100.  Acceptancy scores: Participants ranked 2 constructs the highest “Behavioural intention to use” 5.5 ± 1.1(Mean ± SD) of 7, and “Perceived usefulness” 5.0 ± 1.7(Mean ± SD) of 7, and the other 2 constructs lowest “Intrinsic motivation” 4.3 ± 1.6 (Mean ± SD) of 7, and “Perceived ease of use” 4.7 ± 1.7 of 7(Mean ± SD).  The participants requested additional food items in the food database and some additional functions such as the ability to modify input data, extra entry slots for a snack, to show previous input and a louder alert sound. |
| Johnson [67] | To assess acceptability and satisfaction of daily GDM text messaging system and to refine study procedure and intervention materials. | Base line survey and post intervention questionnaire/ N/A | Post intervention:  Majority of women agreed or strongly agreed that the reminder text messages helped them to take medication, record their blood glucose values, and eat healthier. Majority of women liked the text messages that included information or being a reminder. |
| Khalil [66] | To explore perspectives of women with GDM and HPs about the factors that impact on the adaption and diffusion of myDiabby tele monitoring solution in a context that tele monitoring activities are still not compensated like traditional follow-ups. | N/A / Interview | Women and HPs found some advantages in using myDiabby such as improved patient care like providing regular follow-ups, saving women’s time, reducing women’s traveling, reducing women’s anxiety, empowering women in GDM self-management, and increasing reassurance of having better monitoring by HPs. HPs believed that telemedicine is useful for women who live in a rural area and have to travel long hours to attend in-person visits in healthcare centres. It also helped HPs to interact more often with women. However, some HPs and women believed that there is a need to have an in-person clinical visit.  Women and HPs’ found myDiabby easy and intuitive to use. Due to having positive experiences with using myDiabby, encouraged both women and HPs towards its adoption. The observed benefits of myDiabby helped to adopt the system by the other HPs and also to recommend the system to others by women. The disproportion of HPs numbers to the number of women with GDM encourages the use of telemonitoring. |
| Mackillop [43] | To find out whether managing women with GDM remotely with a GDM mobile real time monitoring blood glucose app is as effective as standard care in controlling blood glucose of women. | Clinical outcomes and patient satisfaction/ Free text question | Both intervention and control groups reported high satisfaction levels for the care that they received.  Majority of participants in both groups reported satisfaction with the number of clinical visits (80% in the control group and 88% in the intervention group). Most of the participants in the intervention group (57 out of 60 women) indicated they would use the app again, and majority of participants in the control group (51 out of 60) also stated they would consider using a mobile phone app.  Almost all participants in the intervention group (59 out of 60 women) mentioned that they would recommend the app to friends or family with the same condition.  In the free-text comments, participants reported the benefits of using GDM such as being convenient, avoiding the appointments at the hospital, and receiving additional support outside of the hospital. |
| Miremberg [21] | To assess the impact of the GDM mobile based system on women’s compliance, blood glucose control, pregnancy outcome, and women satisfaction. | Clinical outcomes and patient satisfaction / N/A | All participants reported “high” or “very high” satisfaction with the application.  80% of the patients reported not having any difficulty using the application.  20% of the patients reported a slight difficulty mainly related to the English language barrier. |
| Nicholson [68] | To refine the GDM management system and to evaluate the intervention feasibility. | Clinical and health information captured by the GDM system.  / Interview and focus group | Ease of use of website: Majority of participants (8 out of 10) found that the website was user-friendly and also it was easy to access.  Participants found the most useful parts of the website were healthy recipes and the tracing of your progress (recording daily weights, exercise, and blood glucose levels).  Despite having little or no experience with an online discussion group, participants favored online peer support such as a message board to share and exchange information with other women with GDM.  Participants were satisfied with the current features. But they had some suggestions such as guidance for the menu of major restaurants to choose healthy food, and also health care professionals to have access to the site. |
| Pais [72] | To document the development of GDM ecosystem and to evaluate  its usefulness and ease of use. | N/A / Interview | Usefulness: Sharing women’s data with health care professionals, combining data from different sources, allowing mothers to manage their condition by themselves, remotely manage mothers, and decreasing costs. Most women preferred to record their data electronically than use paper-based documents.  Ease of use: Summarize data in one screen, easy to navigate, and current experience with mobile app.  Clinicians wanted to record patients’ data into the ecosystem database by themselves. They did not trust the accuracy of the data provided by the patients. |
| Peleg [69] | To document the development of the patient-centered mobile decision-support system and to evaluate its feasibility and its potential effects on healthcare providers and patients. | Satisfaction survey / N/A | Majority of women ranked highest on all satisfaction questions (between 4 and 5 out of 5). Most women answered “Yes” with regard to “Recommending the system to a friend”, and “Continuing to use the system”. In contrast, 12 out of 15 women answered “No” to paying for using the system.  The majority of clinician ranked highest the system as being “useful for managing patients”, “data quality-aware features increase the safety of outpatients” and ranked lowest the system as “increases my overall productivity”, “manage patients in a timely manner using MobiGuide” and “makes it quicker to manage patients”. |
| Peleg[70] | To document the system’s architecture and the developed knowledge elicitation and specification methodologies for enabling personalization and CIGs patient-centered system. It also assessed the feasibility of the system by approaching 3 hypotheses in AF and GDM medical domines. | Questionnaires, Gathering data related to patients and healthcare professionals’ usage pattern/ Interview | Majority of women ranked highest on all satisfaction questions (between 4 and 5 out of 5). Within 17 participants (women with GDM), majority of them (11 out of 17) rated satisfaction with system response time 3 out of 5, and for experiencing errors with the system 9 women rated 3 and under and for helping to visualize and interpret data 6 women rated 3 and under.  Most women answered “Yes” with regard to “Recommending the system to a friend”, and “Continuing to use the system”. In contrast, women’s majority answered “No” to paying for using the system. |
| Pustozerov [76] | To document the system’s architecture and to assess the experience of women for using the system by post-study survey. | Questionnaires for measuring usefulness and convenience (app usability) / Open ended questions. | Women gave the highest mark for the app’s usefulness for disease monitoring. Women gave mainly high marks for the app’s convenience but the median value was low remarkably. This shows that there is a huge need for improving app usability. Most of the women reported that the app is more convenient than a logbook.  The technical problem for women: In the desktop version, women had difficulty sending the email with electronic diary attachment to the clinician. There is a need for a more reliable way to exchange data between patients and healthcare professionals. |
| Rasekaba [75] | To explore perceptions of women with GDM, healthcare professionals and IT staff about the acceptability and feasibility of a GDM telehealth and also to identify the profiles of women who accessing GDM care. | Clinical record audit  / Semi-structured interviews | Women and Healthcare professionals reflected that telehealth is a feasible and acceptable alternative to standard care. They thought telehealth can provide important benefits, particularly for women with GDM in rural areas such as access to care, earlier to get control of blood glucose, increase peer support, and reducing disadvantage aspects of attending in-person clinical visits like taking time off work, and traveling and parking costs. Women believed that telehealth would not affect HPs and women’s relationships and the quality of care that they receive.  However, HPs concerned about increasing their workload by using telehealth due to the increased volume of emails, need to be trained to use telehealth, and coordinate telehealth around other appointments. Also, there was a concern about the costs for GDM telehealth services. |
| Skar [65] | To explore women’s experience about using mHealth system called Pregnant+ app to control their GDM condition. | N/A / Interview | Some women found the app helped their confidence and also motivated them to change their behavior. But some women experienced frustration or obsession while using the app. One of the participants did not agree that there is a need for using the app to access the different information. She found it as a burden.  Several participants had technical problems that led some participants not to use the app for recording their blood glucose levels. Most of them had a problem transferring the blood glucose reading to the app automatically.  One participant mentioned that healthcare professionals were not interested in using the app, and they always asked for a logbook for reviewing the records. Therefore, she stopped using the app. So, one of the barriers of using the app was the lack of support from health care professionals. |
| Surendran [63] | To evaluate the GDM app usage behaviour and explore perception of users about their experience of using the app. | Application usage frequency and participants‘ weight and diet information were collected.  /Semi-structured interviews | Majority of women found educational lessons helped them to manage GDM due to being short, visual representation, easy to understand, and having all the necessary information in one place. Half of the women found the most useful lesson was healthy eating due to helping them to remember the healthy foods. The majority of women did not like the diet track feature because they found it difficult to work and also to have a limited food database. But most women reported weight tracking easy to use and graphical display of weight values helped women to interpret the data.  Some women found the automatic messages helpful for GDM management due to creating self-awareness in food choices and motivating them to change their behavior. |
| Varnfield [71] | To evaluate a mHealth system called M♡THer app by women who were first time diagnosed with GDM. | User satisfaction survey, and number and frequency of uploading blood glucose level using mobile app / Two open ended questions | User satisfaction Results:  Majority of women strongly agreed or agreed that the GDM app helped record their blood glucose level, manage their GDM condition, and provide better support from the health care team. All clinicians strongly agreed or agreed that the app improved their efficiency in providing care to their patients.  Open-ended questions Results:  Women reported some benefits of the app such as convenience, ease of use, ability to monitor their own BGLs, and easy access due to not having to take a paper diary with them. They also liked the quick connection and responses from their doctors.  Women also reported some negative feedback. The main one was technological problems experienced within the app or connecting the app to the blood glucose meter via Bluetooth. Two participants indicated that despite using the app to record their blood glucose readings, the clinical care team still preferred to use a paper diary during in-person clinical visits. |
| Wickramasingh [48] | To evaluate usability, acceptability, and functionality of a mHealth solution. This would lead to generate hypotheses to be  tested in a large-scale in clinical trial. | It is mentioned that the clinical data was collected but they are not available in this paper. / Patient Perspective measured by 4 sets of open ended questionnaires (at the start of project, at the end of the standard care, at the end of the standard care and technology and also at the end of project) and interview. | They mentioned that standard care is difficult and inconvenient. All patients preferred technology solution. However, patients gave some feedback about the lack of information or functions on the mobile solution. For example, including additional information such as diet, the system allows to review previous results and comments and also provides alert notice for the records that are out of the ideal range.  Another patient mentioned it is difficult to carry a logbook with her in different places and it consumes too much time and effort to use it.  All clinicians prefer technology solution. 60% were totally happy and 40% believed it can be improved.  The clinician agreed that technology helped to save time and the efficiency of monitoring women with GDM. Furthermore, providing better comprehensively controlling of blood glucose levels and their changes.  The two diabetes clinicians were concerned about the communication through mobile technology compare to face-to-face clinical visits.  There were some suggestions with regard to the usability of the system for example to distinguish the degree of BG values being out of normal range. |

The references are consistent with the article’s references.

13. Jo S, Park H. Development and evaluation of a smartphone application for managing gestational diabetes mellitus. Healthc Inform Res 2016 Jan;22(1):11-21 [FREE Full text] [doi: 10.4258/hir.2016.22.1.11] [Medline: 26893946]

21. Miremberg H, Ben-Ari T, Betzer T, Raphaeli H, Gasnier R, Barda G, et al. The impact of a daily smartphone-based feedback system among women with gestational diabetes on compliance, glycemic control, satisfaction, and pregnancy outcome: a randomized controlled trial. Am J Obstet Gynecol 2018 Apr;218(4):453.e1-453.e7. [doi: 10.1016/j.ajog.2018.01.044] [Medline: 29425836]

25. Given JE, Bunting BP, O'Kane MJ, Dunne F, Coates VE. Tele-Mum: a feasibility study for a randomized controlled trial exploring the potential for telemedicine in the diabetes care of those with gestational diabetes. Diabetes Technol Ther 2015 Dec;17(12):880-888. [doi: 10.1089/dia.2015.0147] [Medline: 26394017]

27. Harrison TN, Sacks DA, Parry C, Macias M, Ling Grant DS, Lawrence JM. Acceptability of virtual prenatal visits for women with gestational diabetes. Womens Health Issues 2017;27(3):351-355. [doi: 10.1016/j.whi.2016.12.009] [Medline: 28153743]

30. Hirst JE, Mackillop L, Loerup L, Kevat DA, Bartlett K, Gibson O, et al. Acceptability and user satisfaction of a smartphone-based, interactive blood glucose management system in women with gestational diabetes mellitus. J Diabetes Sci Technol 2015 Jan;9(1):111-115 [FREE Full text] [doi: 10.1177/1932296814556506] [Medline: 25361643]

34. Caballero-Ruiz E, García-Sáez G, Rigla M, Villaplana M, Pons B, Hernando ME. A web-based clinical decision support system for gestational diabetes: automatic diet prescription and detection of insulin needs. Int J Med Inform 2017 Jun;102:35-49. [doi: 10.1016/j.ijmedinf.2017.02.014] [Medline: 28495347]

41. Carolan-Olah M, Sayakhot P. A randomized controlled trial of a web-based education intervention for women with gestational diabetes mellitus. Midwifery 2019 Jan;68:39-47. [doi: 10.1016/j.midw.2018.08.019] [Medline: 30343264]

43. Mackillop L, Hirst JE, Bartlett KJ, Birks JS, Clifton L, Farmer AJ, et al. Comparing the efficacy of a mobile phone-based blood glucose management system with standard clinic care in women with gestational diabetes: randomized controlled trial. JMIR Mhealth Uhealth 2018 Mar 20;6(3):e71 [FREE Full text] [doi: 10.2196/mhealth.9512] [Medline: 29559428]

48. Wickramasinghe N, Gururajan R. Innovation practice using pervasive mobile technology solutions to improve population health management: a pilot study of gestational diabetes patient care in Australia. J Healthc Qual 2016;38(2):93-105. [doi: 10.1097/JHQ.0000000000000033] [Medline: 26918811]

50. Bartholomew ML, Soules K, Church K, Shaha S, Burlingame J, Graham G, et al. Managing diabetes in pregnancy using cell phone/internet technology. Clin Diabetes 2015 Oct;33(4):169-174 [FREE Full text] [doi: 10.2337/diaclin.33.4.169] [Medline: 26487790]

62. Garnweidner-Holme L, Hoel Andersen T, Sando MW, Noll J, Lukasse M. Health care professionals' attitudes toward, and experiences of using, a culture-sensitive smartphone app for women with gestational diabetes mellitus: qualitative study. JMIR Mhealth Uhealth 2018 May 14;6(5):e123 [FREE Full text] [doi: 10.2196/mhealth.9686] [Medline: 29759959]

63. Surendran S, Lim CS, Koh GC, Yew TW, Tai ES, Foong PS. Women's usage behavior and perceived usefulness with using a mobile health application for gestational diabetes mellitus: mixed-methods study. Int J Environ Res Public Health 2021 Jun 21;18(12):6670 [FREE Full text] [doi: 10.3390/ijerph18126670] [Medline: 34205744]

64. Bromuri S, Puricel S, Schumann R, Krampf J, Ruiz J, Schumacher M. An expert personal health system to monitor patients affected by gestational diabetes mellitus: a feasibility study. J Ambient Intelligence Smart Environ 2016 Mar 15;8(2):219-237. [doi: 10.3233/ais-160365]

65. Skar JB, Garnweidner-Holme LM, Lukasse M, Terragni L. Women's experiences with using a smartphone app (the Pregnant+ app) to manage gestational diabetes mellitus in a randomised controlled trial. Midwifery 2018 Mar;58:102-108. [doi: 10.1016/j.midw.2017.12.021] [Medline: 29329023]

66. Khalil C. Understanding the adoption and diffusion of a telemonitoring solution in gestational diabetes mellitus: qualitative study. JMIR Diabetes 2019 Nov 28;4(4):e13661 [FREE Full text] [doi: 10.2196/13661] [Medline: 31778118]

67. Johnson QB, Berry DC. Impacting diabetes self-management in women with gestational diabetes mellitus using short messaging reminders. J Am Assoc Nurse Pract 2018 Jun;30(6):320-326. [doi: 10.1097/JXX.0000000000000059] [Medline: 29878964]

68. Nicholson WK, Beckham AJ, Hatley K, Diamond M, Johnson L, Green SL, et al. The Gestational Diabetes Management System (GooDMomS): development, feasibility and lessons learned from a patient-informed, web-based pregnancy and postpartum lifestyle intervention. BMC Pregnancy Childbirth 2016 Sep 21;16(1):277 [FREE Full text] [doi:

10.1186/s12884-016-1064-z] [Medline: 27654119]

69. Peleg M, Shahar Y, Quaglini S, Broens T, Budasu R, Fung N, et al. Assessment of a personalized and distributed patient guidance system. Int J Med Inform 2017 May;101:108-130. [doi: 10.1016/j.ijmedinf.2017.02.010] [Medline: 28347441]

70. Peleg M, Shahar Y, Quaglini S, Fux A, García-Sáez G, Goldstein A, et al. MobiGuide: a personalized and patient-centric decision-support system and its evaluation in the atrial fibrillation and gestational diabetes domains. User Model User-Adap Inter 2017 Mar 11;27(2):159-213. [doi: 10.1007/s11257-017-9190-5]

71. Varnfield M, Redd C, Stoney RM, Higgins L, Scolari N, Warwick R, et al. M♡THer, an mHealth system to support women with gestational diabetes mellitus: feasibility and acceptability study. Diabetes Technol Ther 2021 May;23(5):358-366 [FREE Full text] [doi: 10.1089/dia.2020.0509] [Medline: 33210954]

72. Pais S, Parry D, Petrova K, Rowan J. Acceptance of using an ecosystem of mobile apps for use in diabetes clinic for self-management of gestational diabetes mellitus. Stud Health Technol Inform 2017;245:188-192. [Medline: 29295079]

73. Edwards KJ, Bradwell HL, Jones RB, Andrade J, Shawe JA. How do women with a history of gestational diabetes mellitus use mHealth during and after pregnancy? Qualitative exploration of women's views and experiences. Midwifery 2021 Jul;98:102995. [doi: 10.1016/j.midw.2021.102995] [Medline: 33784541]

74. Gianfrancesco C, Darwin Z, McGowan L, Smith DM, Haddrill R, Carter M, et al. Exploring the feasibility of use of an online dietary assessment tool (myfood24) in women with gestational d.iabetes. Nutrients 2018 Aug 23;10(9):1147 [FREE Full text] [doi: 10.3390/nu10091147] [Medline: 30142898]

75. Rasekaba T, Nightingale H, Furler J, Lim WK, Triay J, Blackberry I. Women, clinician and IT staff perspectives on telehealth for enhanced gestational diabetes mellitus management in an Australian rural/regional setting. Rural Remote Health 2021 Jan;21(1):5983 [FREE Full text] [doi: 10.22605/RRH5983] [Medline: 33478229]

76. Pustozerov E, Popova P. Mobile-based decision support system for gestational diabetes mellitus. In: Proceedings of the 2018 Ural Symposium on Biomedical Engineering, Radioelectronics and Information Technology (USBEREIT). 2018 Presented at: 2018 Ural Symposium on Biomedical Engineering, Radioelectronics and Information Technology (USBEREIT); May 07-08, 2018; Yekaterinburg, Russia. [doi: 10.1109/usbereit.2018.8384546]

77. Garnweidner-Holme LM, Borgen I, Garitano I, Noll J, Lukasse M. Designing and developing a mobile smartphone application for women with gestational diabetes mellitus followed-up at diabetes outpatient clinics in Norway. Healthcare (Basel) 2015

May 21;3(2):310-323 [FREE Full text] [doi: 10.3390/healthcare3020310] [Medline: 27417764]
