# Supplementary material for: Barriers to and Facilitators of Using eHealth to Support Gestational Diabetes Mellitus Self-management: Systematic Literature Review of Perceptions of Health Care Professionals and Women With Gestational Diabetes Mellitus
Source: J Med Internet Res. 2022 Oct 27;24(10):e39689. doi: 10.2196/39689 (PMC9650580; doi:10.2196/39689)
Supplement: Multimedia Appendix 3 [file jmir_v24i10e39689_app3.docx]

Appendix 3: Quality assessment

Quality assessment for quantitative methodology (questionnaire or survey)

| **Reference:** First Author | Peleg [69] | Johnson [67] |
| --- | --- | --- |
| **Research question and study design** | |  |
| Was a questionnaire the most appropriate method? | It was appropriate but it could be followed with open ended question or interview for get data in depth. | It was appropriate but it could be followed with open ended question or interview for get data in depth. |
| **Validity and reliability** | |  |
| Have claims for validity been made, and are they justified? (Is there evidence that the instrument measures what it sets out to measure?) | There is no information available for construct validity of the questionnaire. | There is no information available for construct validity of the questionnaire. |
| Have claims for reliability been made, and are they justified? (Is there evidence that the questionnaire provides stable responses over time and between researchers?) | There is no information available for reliability (stability or internal consistency) of the questionnaire. | There is no information available for reliability (stability or internal consistency) of the questionnaire |
| **Format** | |  |
| Are example questions provided? | Yes | Yes |
| Did the questions make sense, and could the participants in the sample understand them? Were any questions ambiguous or overly complicated? | The questions make sense, understandable and they are not complicated. But there is no information available in the paper about view of participants with regard to perception of the questions. | It makes sense and understandable. |
| **Piloting** | |  |
| Are details given about the piloting undertaken |  |  |
| Was the questionnaire adequately piloted in terms of the method and means of administration, on people who were representative of the study population? | There is no information available with regard to pilot the questionnaire. | There is no information available with regard to pilot the questionnaire. |
| **Sampling** | | |
| Was the sampling frame for the definitive study sufficiently large and representative? | The sample is small and the size of sample is not justified. There is no more information available. | The sample size was small just 19 women with GDM. Therefore, the results are not generalizable. The participants also were not representative because they were chosen based on having ability to read and write and owing a mobile phone |
| **Distribution, administration and response** | |  |
| Was the method of distribution and administration reported | Participants with GDM (19) and healthcare professional (6) answered the questionnaire at the end of study. There is no more information available. | The survey distributed via the electronic medical record (EPIC) MyChart. |
| Were the response rates reported, including details of participants who were unsuitable for the research or refused to take part? | All participants and healthcare professional completed the questionnaire at the end of study. But there is no information available about the respond rate with regard to GDM population or about inclusion and exclusion criteria. | Among 27 people, 3 women never register for the electronic medical record therefore did not have access to survey link, 3 other women did not completed the survey and 2 women delivered before completing the initial  demographic information. Therefore 19 women participated for this study. |
| Have any potential response biases been discussed? | N/A | There is no information available. |
| **Coding and analysis** | | |
| What sort of analysis was carried out and was this appropriate? (e.g. correct statistical tests for quantitative | T test has been used for clinical outcomes results.  The questionnaire was answered on Likert scale of 1 to 5. But there is no more information available. | Descriptive statistic. It was appropriate. |
| answers, qualitative analysis for open ended questions) | N/A | N/A |
| **Results** | | |
| Were all relevant data reported? | Yes. | Yes |
| Are quantitative results definitive (significant), and are relevant non-significant results also reported? | For clinical outcomes significant and non-significant results reported.  Questionnaire results have been analysed based on the number of users who ranked the variables on Likert scale of 1 through 5. | Questionnaire results have been analysed based on the number of users who ranked the variables on Likert scale of strongly disagree, disagree, Neither agree nor disagree, agree and strongly agree. |
| Have qualitative results been adequately interpreted (e.g. using an explicit theoretical framework), and have any quotes been properly justified and contextualised? | N/A | N/A |
| **Conclusions and discussion** | |  |
| Have the researchers drawn an appropriate link between the data and their conclusions? | Yes | Yes |
| Have the findings been placed within the wider body of knowledge in the field (e.g. via a comprehensive literature review), and are any recommendations justified? | Their findings were supported by some literature review in the field but It is not comprehensive. | Yes |

Quality assessment for qualitative methodology.

| Reference: First Author | Edwards [73], Garnweidner-Holme [62], Khalil [66], Pais [72], Skar [65]. | | | |
| --- | --- | --- | --- | --- |
|  | Responses | | | |
|  | Yes | No | Can’t tell | comment |
| **Section A: Are the results valid?** |  |  |  |  |
| 1. Was there a clear statement of the aims of the research? | Edwards [73], Garnweidner-Holme [62], Khalil [66], Pais [72], Skar [65]. |  |  |  |
| 2. Is a qualitative methodology appropriate? | Edwards [73], Garnweidner-Holme [62], Khalil [66], Pais [72], Skar [65]. |  |  |  |
| **Is it worth continuing?** |  |  |  |  |
| 3. Was the research design appropriate to address the aims of the research? | Edwards [73], Garnweidner-Holme [62], Khalil [66], Pais [72], Skar [65]. |  |  |  |
| 4. Was the recruitment strategy appropriate to the aims of the research? | Edwards [73], Garnweidner-Holme [62], Khalil [66], Skar [65]. |  | Pais [72]. | Inclusion and exclusion criteria were not mentioned in this paper Pais [72]. |
| 5. Was the data collected in a way that addressed the research issue? | Edwards [73], Garnweidner-Holme [62], Khalil [66], Pais [72], Skar [65]. |  |  | The first stage was explained clearly that how they collected data from clinicians but in the next part that the system reviewed by women with GDM there is no information of how the data gathered Pais [72] |
| 6. Has the relationship between researcher and participants been adequately considered? | Edwards [73]. |  | Garnweidner-Holme [62], Khalil [66], Pais [72], Skar [65]. | There is no information available Garnweidner-Holme [62], Khalil [66], Pais [72], Skar [65]. |
| **Section B: What are the results?** |  |  |  |  |
| 7. Have ethical issues been taken into consideration? | Edwards [73], Khalil [66], Skar [65]. |  | Garnweidner-Holme [62], Pais [72]. | There is no information available in the paper with regard to ethical issues Pais [72]. |
| 8. Was the data analysis sufficiently rigorous? | Edwards [73], Garnweidner-Holme [62], Khalil [66], Skar [65]. |  | Pais [72]. | There is no information available about analysis process in the paper. It just included summary of the finding Pais [72]. |
| 9. Is there a clear statement of findings? | Edwards [73], Garnweidner-Holme [62], Khalil [66], Pais [72], Skar [65]. |  |  |  |
| 10. How valuable is the research? | The paper Pais [72] explained the importance of contribution of this project with existing technology in order to improve the health care quality with sharing data and involving women with GDM actively in the health care process. The paper Skar [65] explored the possibility of using m-health to help women in order to self-management of GDM and the potential challenge of using m-health. This findings are valuable in order to improve using m-health technology that address these challenges. Garnweidner-Holme [62] study is valuable because it explored the opinion of healthcare professionals about their experience of providing care to women with GDM through mHealth. Khalil [66] study is valuable because its results showed women and HPs’ opinions about their experience in using a telemedicine solution for GDM management and why telemedicine is not remunerated as traditional healthcare method. Edwards [73] is a valuable research due to exploring women’s perspectives about using mhealth to manage GDM condition. | | | |

Quality assessment for mixed methods.

| Reference: First Author | Bromuri [64], Carolan-Olah [41], Garnweidner-Holme [77], Gianfrancesco [74], Given [25], Harrison [27], Hirst [30], Jo [13], Mackillop [43], Nicholson [68], Pustozerov [76], Rasekaba [75], Surendran [63], Varnfield [71], Wickramasingh [48] and Peleg [70]. | | | | |
| --- | --- | --- | --- | --- | --- |
| Types of mixed methods study components or primary studies | Methodological quality criteria | Responses | | | |
|  |  | Yes | No | Can’t  tell | Comments |
| Screening questions (for all types) | Are there clear qualitative and quantitative research questions (or objectives*), or a clear mixed methods question (or objective*)? | Bromuri [64], Carolan-Olah [41], Garnweidner-Holme [77], Gianfrancesco [74], Given [25], Harrison [27], Hirst [30], Jo [13], Mackillop [43], Nicholson [68], Pustozerov [76], Rasekaba [75], Surendran [63], Varnfield [71], and Wickramasingh [48], Peleg [70]. |  |  |  |
|  | Do the collected data allow address the research question (objective)? E.g., consider whether the follow-up period is long enough for the outcome to occur (for longitudinal studies or study components). | Bromuri [64], Carolan-Olah [41], Garnweidner-Holme [77], Gianfrancesco [74], Given [25], Harrison [27], Hirst [30], Jo [13], Mackillop [43], Nicholson [68], Pustozerov [76], Rasekaba [75], Surendran [63], Varnfield [71], and Wickramasingh [48], Peleg [70]. |  |  |  |
|  | Further appraisal may be not feasible or appropriate when the answer is ‘No’ or ‘Can’t tell’ to one or both screening questions | | | | |
| 1. Qualitative | 1.1. Are the sources of qualitative data (archives, documents, informants, observations) relevant to address the research question (objective)? | Bromuri [64], Carolan-Olah [41], Garnweidner-Holme [77], Gianfrancesco [74], Given [25], Harrison [27], Hirst [30], Jo [13], Mackillop [43], Nicholson [68], Pustozerov [76], Rasekaba [75], Surendran [63], Varnfield [71], Peleg [70] , and Wickramasingh [48]. |  |  |  |
|  | 1.2. Is the process for analysing qualitative data relevant to address the research question (objective)? | Garnweidner-Holme [77], Gianfrancesco [74], Given [25], Harrison [27], Hirst [30], Mackillop [43], Nicholson [68], Pustozerov [76], Rasekaba [75], Surendran [63], Varnfield [71], Peleg [70] , and Wickramasingh [48]. |  | Bromuri [64], and Carolan- Carolan-Olah [41], Jo [13]. | There is no information about how qualitative data was analysed (Carolan-Olah [41]. The method of gathering data was open ended survey but there is no information available for analysing the qualitative data Jo [13]. |
|  | 1.3. Is appropriate consideration given to how findings relate to the context, e.g., the setting, in which the data were collected? | Carolan-Olah [41], Harrison [27], Hirst [30], Mackillop [43], Rasekaba [75], Surendran [63], Varnfield [71], Peleg [70] and Wickramasingh [48]. | Gianfrancesco [74], and Pustozerov [76] | Bromuri [64], Carolan-Olah [41], Garnweidner-Holme [77], Given [25], Jo [13], and Nicholson [68]. | There is no information available Bromuri [64], Carolan-Olah [41], Garnweidner-Holme [77], Given [25], Jo [13], and Nicholson [68]. |
|  | 1.4. Is appropriate consideration given to how findings relate to researchers’ influence, e.g., through their interactions with participants? | Carolan-Olah [41], | Gianfrancesco [74], and Pustozerov [76] | Bromuri [64], Garnweidner-Holme [77], Given [25], Harrison [27], Hirst [30], Jo [13], Mackillop [43], Nicholson [68], Rasekaba [75], Surendran [63], Varnfield [71], Peleg [70] , and Wickramasingh [48]. | There is no information available Bromuri [64], Garnweidner-Holme [77], Given [25], Harrison [27], Hirst [30], Jo [13], Mackillop [43], Nicholson [68], Rasekaba [75], Surendran [63], Varnfield [71], Plege [78], and Wickramasingh [48]. |
| 2. Quantitative randomized controlled (trials) | 2.1. Is there a clear description of the randomization (or an appropriate sequence generation)? | Bromuri [64], Given [25], and Mackillop [43]. |  |  |  |
|  | 2.2. Is there a clear description of the allocation concealment (or blinding when applicable)? | Given [25] |  | Bromuri [64], Mackillop [43]. | Open label Bromuri [64]. |
|  | 2.3. Are there complete outcome data (80% or above)? | Bromuri [64], Given [25], and Mackillop [43]. |  |  |  |
|  | 2.4. Is there low withdrawal/drop-out (below 20%)? | Bromuri [64], Given [25], and Mackillop [43]. |  |  |  |
| 3. Quantitative nonrandomized | 3.1. Are participants (organizations) recruited in a way that minimizes selection bias? | Wickramasingh [48]. |  |  |  |
|  | 3.2. Are measurements appropriate (clear origin, or validity known, or standard instrument; and absence of contamination between groups when appropriate) regarding the exposure/intervention and outcomes? |  |  | Wickramasingh [48]. | patients have to complete an open-ended questionnaires four times in different stages of study with different purposes but there is no information about that what was the source of these questionnaire or how they developed them and also about the validity of these questionnaires. The questions are not included in the paper. |
|  | 3.3. In the groups being compared (exposed vs. non-exposed; with intervention vs. without; cases vs. controls), are the participants comparable, or do researchers take into account (control for) the difference between these groups? |  | Wickramasingh [48]. |  |  |
|  | 3.4. Are there complete outcome data (80% or above), and, when applicable, an acceptable response rate (60% or above), or an acceptable follow-up rate for cohort studies (depending on the duration of follow-up)? | Wickramasingh [48]. |  |  | Only the qualitative data is available in this study. The quantitative data of this study related to patient outcomes (for example: use of insulin, compliance with blood monitoring, delivery outcome) is not available on the paper. |
| 4. Quantitative descriptive | 4.1. Is the sampling strategy relevant to address the quantitative research question (quantitative aspect of the mixed methods question)? | Carolan-Olah [41], Garnweidner-Holme [77], Gianfrancesco [74], Given [25], Nicholson [68], Pustozerov [76], Rasekaba [75], Surendran [63], and Varnfield [71], Peleg [70]. |  | Harrison [27], Hirst [30], and Jo [13], | The sample size is not justified Hirst [30]. The sample size was small for survey (70) and participants were just from one clinics Harrison [27]. The sample was from relevant population (GDM women) but there is no evidence of justifying the sample size in the paper Jo [13]. |
|  | 4.2. Is the sample representative of the population understudy? | Carolan-Olah [41], Garnweidner-Holme [77], Gianfrancesco [74], Given [25], Harrison [27], Hirst [30], Jo [13], Nicholson [68],Pustozerov [76], Rasekaba [75], Surendran [63], and Varnfield [71], Peleg [70]. |  |  |  |
|  | 4.3. Are measurements appropriate (clear origin, or validity known, or standard instrument)? | Carolan-Olah [41], Garnweidner-Holme [77], Gianfrancesco [74], Harrison [27], Hirst [30], Jo [13], Nicholson [68], Pustozerov [76], Rasekaba [75], and Surendran [63]. |  | Given [25], Peleg [70] and Varnfield [71] | Adapted version of satisfaction questionnaire was used and the references were provided to show the permission of the adaption. But the questions or measurements were not included in the study and also the proof of validation of this adaption were not considered Given [25].  No information about validity or reliability of the satisfaction questionnaire is available. There is no information available about how the satisfactions questionnaire has been developed Varnfield [71] and Peleg [70]. |
|  | 4.4. Is there an acceptable response rate (60% or above)? | Carolan-Olah [41], Garnweidner-Holme [77], Given [25], Harrison [27], Hirst [30], Nicholson [68], Rasekaba [75], Surendran [63], Peleg [70], and Varnfield [71]. | Jo [13], and Gianfrancesco [74] | Pustozerov [76] | 22 out of 60 were included in analysis Jo [13].  73 out of 199 answered the questionnaire Gianfrancesco [74]. |
| 5. Mixed methods | 5.1. Is the mixed methods research design relevant to address the qualitative and quantitative research questions (or objectives), or the qualitative and quantitative aspects of the mixed methods question (or objective)? | Bromuri [64], Carolan-Olah [41], Garnweidner-Holme [77], Gianfrancesco [74], Given [25], Hirst [30], Jo [13], Mackillop [43], Nicholson [68], Pustozerov [76], Rasekaba [75], Surendran [63], Varnfield [71], Peleg [70], and Wickramasingh [48]. |  | Harrison [27] | The rational for integrating quantitative and qualitative data was not explained Harrison [27]. |
|  | 5.2. Is the integration of qualitative and quantitative data (or results*) relevant to address the research question (objective)? | Bromuri [64], Carolan-Olah [41], Garnweidner-Holme [77], Gianfrancesco [74], Given [25], Hirst [30], Jo [13], Mackillop [43], Nicholson [68], Pustozerov [76], Rasekaba [75], Surendran [63], Peleg [70] , and Varnfield [71]. |  | Wickramasinghe [48], and Harrison [27]. | The questions (open ended) are not included in the paper Wickramasinghe [48] but the results are mainly qualitative data. The quantitative data is not available in this paper but in the small paragraph just mentioned to few figures like percentage of satisfaction of health care professional about technology. So, it is difficult to say the integration of qualitative and quantitative data is relevant to research question Wickramasinghe [48]. The quantitative data related to survey were not provided in the paper, only the themes that created from qualitative data from survey was mentioned. There is no information about the integration data Harrison [27]. |
|  | 5.3. Is appropriate consideration given to the limitations associated with this integration, e.g., the divergence of qualitative and quantitative data (or results*) in a triangulation design? | Wickramasinghe [48] and Peleg [70]. | Pustozerov [76], Gianfrancesco [74], and Rasekaba [75] | Bromuri [64], Carolan-Olah [41], Garnweidner-Holme [77], Harrison [27], Hirst [30], Jo [13], Mackillop [43], Nicholson [68], Surendran [63], and Varnfield [71]. | There is no information available: Bromuri [64], Carolan-Olah [41], Garnweidner-Holme [77], Harrison [27], Hirst [30], Jo [13], Mackillop [43], Nicholson [68], Surendran [63] and Varnfield [71]. |
|  | Criteria for the qualitative component (1.1 to 1.4), and appropriate criteria for the quantitative component (2.1 to 2.4, or 3.1 to 3.4, or 4.1 to 4.4), must be also applied. | | | | |

The references are consistent with the article’s references.

13. Jo S, Park H. Development and evaluation of a smartphone application for managing gestational diabetes mellitus. Healthc

Inform Res 2016 Jan;22(1):11-21 [FREE Full text] [doi: 10.4258/hir.2016.22.1.11] [Medline: 26893946]

21. Miremberg H, Ben-Ari T, Betzer T, Raphaeli H, Gasnier R, Barda G, et al. The impact of a daily smartphone-based feedback

system among women with gestational diabetes on compliance, glycemic control, satisfaction, and pregnancy outcome: a

randomized controlled trial. Am J Obstet Gynecol 2018 Apr;218(4):453.e1-453.e7. [doi: 10.1016/j.ajog.2018.01.044]

[Medline: 29425836]

25. Given JE, Bunting BP, O'Kane MJ, Dunne F, Coates VE. Tele-Mum: a feasibility study for a randomized controlled trial

exploring the potential for telemedicine in the diabetes care of those with gestational diabetes. Diabetes Technol Ther 2015

Dec;17(12):880-888. [doi: 10.1089/dia.2015.0147] [Medline: 26394017]

27. Harrison TN, Sacks DA, Parry C, Macias M, Ling Grant DS, Lawrence JM. Acceptability of virtual prenatal visits for

women with gestational diabetes. Womens Health Issues 2017;27(3):351-355. [doi: 10.1016/j.whi.2016.12.009] [Medline:

28153743]

30. Hirst JE, Mackillop L, Loerup L, Kevat DA, Bartlett K, Gibson O, et al. Acceptability and user satisfaction of a

smartphone-based, interactive blood glucose management system in women with gestational diabetes mellitus. J Diabetes

Sci Technol 2015 Jan;9(1):111-115 [FREE Full text] [doi: 10.1177/1932296814556506] [Medline: 25361643]

34. Caballero-Ruiz E, García-Sáez G, Rigla M, Villaplana M, Pons B, Hernando ME. A web-based clinical decision support

system for gestational diabetes: automatic diet prescription and detection of insulin needs. Int J Med Inform 2017

Jun;102:35-49. [doi: 10.1016/j.ijmedinf.2017.02.014] [Medline: 28495347]

41. Carolan-Olah M, Sayakhot P. A randomized controlled trial of a web-based education intervention for women with gestational

diabetes mellitus. Midwifery 2019 Jan;68:39-47. [doi: 10.1016/j.midw.2018.08.019] [Medline: 30343264]

43. Mackillop L, Hirst JE, Bartlett KJ, Birks JS, Clifton L, Farmer AJ, et al. Comparing the efficacy of a mobile phone-based

blood glucose management system with standard clinic care in women with gestational diabetes: randomized controlled

trial. JMIR Mhealth Uhealth 2018 Mar 20;6(3):e71 [FREE Full text] [doi: 10.2196/mhealth.9512] [Medline: 29559428]

48. Wickramasinghe N, Gururajan R. Innovation practice using pervasive mobile technology solutions to improve population

health management: a pilot study of gestational diabetes patient care in Australia. J Healthc Qual 2016;38(2):93-105. [doi:

10.1097/JHQ.0000000000000033] [Medline: 26918811]

50. Bartholomew ML, Soules K, Church K, Shaha S, Burlingame J, Graham G, et al. Managing diabetes in pregnancy using

cell phone/internet technology. Clin Diabetes 2015 Oct;33(4):169-174 [FREE Full text] [doi: 10.2337/diaclin.33.4.169]

[Medline: 26487790]

62. Garnweidner-Holme L, Hoel Andersen T, Sando MW, Noll J, Lukasse M. Health care professionals' attitudes toward, and

experiences of using, a culture-sensitive smartphone app for women with gestational diabetes mellitus: qualitative study.

JMIR Mhealth Uhealth 2018 May 14;6(5):e123 [FREE Full text] [doi: 10.2196/mhealth.9686] [Medline: 29759959]

63. Surendran S, Lim CS, Koh GC, Yew TW, Tai ES, Foong PS. Women's usage behavior and perceived usefulness with using

a mobile health application for gestational diabetes mellitus: mixed-methods study. Int J Environ Res Public Health 2021

Jun 21;18(12):6670 [FREE Full text] [doi: 10.3390/ijerph18126670] [Medline: 34205744]

64. Bromuri S, Puricel S, Schumann R, Krampf J, Ruiz J, Schumacher M. An expert personal health system to monitor patients

affected by gestational diabetes mellitus: a feasibility study. J Ambient Intelligence Smart Environ 2016 Mar 15;8(2):219-237.

[doi: 10.3233/ais-160365]

65. Skar JB, Garnweidner-Holme LM, Lukasse M, Terragni L. Women's experiences with using a smartphone app (the Pregnant+

app) to manage gestational diabetes mellitus in a randomised controlled trial. Midwifery 2018 Mar;58:102-108. [doi:

10.1016/j.midw.2017.12.021] [Medline: 29329023]

66. Khalil C. Understanding the adoption and diffusion of a telemonitoring solution in gestational diabetes mellitus: qualitative

study. JMIR Diabetes 2019 Nov 28;4(4):e13661 [FREE Full text] [doi: 10.2196/13661] [Medline: 31778118]

67. Johnson QB, Berry DC. Impacting diabetes self-management in women with gestational diabetes mellitus using short

messaging reminders. J Am Assoc Nurse Pract 2018 Jun;30(6):320-326. [doi: 10.1097/JXX.0000000000000059] [Medline:

29878964]

68. Nicholson WK, Beckham AJ, Hatley K, Diamond M, Johnson L, Green SL, et al. The Gestational Diabetes Management

System (GooDMomS): development, feasibility and lessons learned from a patient-informed, web-based pregnancy and

postpartum lifestyle intervention. BMC Pregnancy Childbirth 2016 Sep 21;16(1):277 [FREE Full text] [doi:

10.1186/s12884-016-1064-z] [Medline: 27654119]

69. Peleg M, Shahar Y, Quaglini S, Broens T, Budasu R, Fung N, et al. Assessment of a personalized and distributed patient

guidance system. Int J Med Inform 2017 May;101:108-130. [doi: 10.1016/j.ijmedinf.2017.02.010] [Medline: 28347441]

70. Peleg M, Shahar Y, Quaglini S, Fux A, García-Sáez G, Goldstein A, et al. MobiGuide: a personalized and patient-centric

decision-support system and its evaluation in the atrial fibrillation and gestational diabetes domains. User Model User-Adap

Inter 2017 Mar 11;27(2):159-213. [doi: 10.1007/s11257-017-9190-5]

71. Varnfield M, Redd C, Stoney RM, Higgins L, Scolari N, Warwick R, et al. M♡THer, an mHealth system to support women

with gestational diabetes mellitus: feasibility and acceptability study. Diabetes Technol Ther 2021 May;23(5):358-366

[FREE Full text] [doi: 10.1089/dia.2020.0509] [Medline: 33210954]

72. Pais S, Parry D, Petrova K, Rowan J. Acceptance of using an ecosystem of mobile apps for use in diabetes clinic for

self-management of gestational diabetes mellitus. Stud Health Technol Inform 2017;245:188-192. [Medline: 29295079]

73. Edwards KJ, Bradwell HL, Jones RB, Andrade J, Shawe JA. How do women with a history of gestational diabetes mellitus

use mHealth during and after pregnancy? Qualitative exploration of women's views and experiences. Midwifery 2021

Jul;98:102995. [doi: 10.1016/j.midw.2021.102995] [Medline: 33784541]

74. Gianfrancesco C, Darwin Z, McGowan L, Smith DM, Haddrill R, Carter M, et al. Exploring the feasibility of use of an

online dietary assessment tool (myfood24) in women with gestational d.iabetes. Nutrients 2018 Aug 23;10(9):1147 [FREE

Full text] [doi: 10.3390/nu10091147] [Medline: 30142898]

75. Rasekaba T, Nightingale H, Furler J, Lim WK, Triay J, Blackberry I. Women, clinician and IT staff perspectives on telehealth

for enhanced gestational diabetes mellitus management in an Australian rural/regional setting. Rural Remote Health 2021

Jan;21(1):5983 [FREE Full text] [doi: 10.22605/RRH5983] [Medline: 33478229]

76. Pustozerov E, Popova P. Mobile-based decision support system for gestational diabetes mellitus. In: Proceedings of the

2018 Ural Symposium on Biomedical Engineering, Radioelectronics and Information Technology (USBEREIT). 2018

Presented at: 2018 Ural Symposium on Biomedical Engineering, Radioelectronics and Information Technology (USBEREIT);

May 07-08, 2018; Yekaterinburg, Russia. [doi: 10.1109/usbereit.2018.8384546]

77. Garnweidner-Holme LM, Borgen I, Garitano I, Noll J, Lukasse M. Designing and developing a mobile smartphone application

for women with gestational diabetes mellitus followed-up at diabetes outpatient clinics in Norway. Healthcare (Basel) 2015

May 21;3(2):310-323 [FREE Full text] [doi: 10.3390/healthcare3020310] [Medline: 27417764]
