# Supplementary material for: Barriers to and Facilitators of Using eHealth to Support Gestational Diabetes Mellitus Self-management: Systematic Literature Review of Perceptions of Health Care Professionals and Women With Gestational Diabetes Mellitus
Source: J Med Internet Res. 2022 Oct 27;24(10):e39689. doi: 10.2196/39689 (PMC9650580; doi:10.2196/39689)
Supplement: Multimedia Appendix 4 [file jmir_v24i10e39689_app4.docx]

Multimedia Appendix 4

Definition of the themes and sub-themes

| Benefits of Using Technology (Theme 1) | This theme included women with GDM and HPs' opinions about benefits and positive effects of using technology regarding GDM management. |
| --- | --- |
| 1.a: Convenience of technology use(Sub-theme) | This sub-theme included women and HPs' opinions about the reasons that GDM systems are convenient to use for managing GDM condition. |
| 1.b: Improving GDM self-management by using technology (Sub-theme) | This sub-theme included women and HPs opinions about how technology could help women to improve their knowledge and ability to manage their GDM condition. |

| Engagement With People via Technology (Theme 2) | This theme included the opinions of women with GDM about communication with other women with GDM. This sub-theme also includes women’s perceptions about technology as a way of communication with healthcare professionals regarding GDM management. Furthermore, it includes the opinions of healthcare professionals about communication with women with GAM with using technology and its benefits and problems. |
| --- | --- |

| Usability of Technology. (Theme 3) | This theme included the opinions of women with GDM and HPs about usability of technologies that they used to manage their GDM. This includes their positive views, their concerns and suggestions to improve the usability of these technologies. |
| --- | --- |

| Discouragement Factors for the Use of Technology (Theme 4) | This theme oriented around the opinions of women and HPs on adverse factors that affect the use of these systems. |
| --- | --- |
