# Supplementary material for: Barriers to and Facilitators of Using eHealth to Support Gestational Diabetes Mellitus Self-management: Systematic Literature Review of Perceptions of Health Care Professionals and Women With Gestational Diabetes Mellitus
Source: J Med Internet Res. 2022 Oct 27;24(10):e39689. doi: 10.2196/39689 (PMC9650580; doi:10.2196/39689)
Supplement: Multimedia Appendix 5 [file jmir_v24i10e39689_app5.docx]

Multimedia Appendix 5: Satisfaction measurement

| First Author | Features | Measurement of satisfaction | Point-scale items |
| --- | --- | --- | --- |
|  |  |  |  |
| Bartholomew [50] | Blood glucose management. | 1) Easy to use; 2) Most enjoyable; 3) Least time consuming; 4) Liked best; 5) Increased motivation for self-management; 6) Recommended to friends and family; 7) Improved diabetes control; 8) Most personolised; 9) Most likely to do fingerstickes?  (Satisfaction survey results of pregnant women using a CIT or voicemail SMBG reporting system in a diabetes management program). | Percentage |
| Caballero-Ruiz [34] | Blood glucose management, ketonuria management, diet management, recording insulin and physical activity, and recommendations for GDM management. | 1) General impression is good; 2) Trust is being well controlled; 3) Satisfaction regarding diabetes follow Up; 4)Using the system avoids displacements; 5) Number of hospital consultations is enough; 6) The system helps in data interpretation; 7) The system helps to improve my GDM knowledge; 8) The patient would recommend the system; 9) The system does not complicate my daily life; 10) No pressure about sending data frequently; 11) Clarity of visualization treatment changes; 12) Ease to learn how to use the System; 13) The application is useful. | 10-point scale |
| Given [25] | Blood glucose, weight and blood pressure management. | Adapted version of the Telemedicine Satisfaction and Usefulness Questionnaire by S. Bakken. | Likert scale |
| Hirst [30] | Blood glucose management, data transmission to a website within the NHS and GDM management messages. | Women’s overall satisfaction with  gestational diabetes care:  I am satisfied with my current treatment; I am satisfied the treatment; I am receiving is the best  for me; I am satisfied with my understanding of diabetes  Relationship with the diabetes clinical care team:  I feel my maternity diabetes team knows enough about my current level of diabetes control; I feel I have a good relationship with my maternity diabetes team; I am satisfied with my maternity diabetes team’s understanding of my diabetes.  Satisfaction with the GDm-health system:  I find the equipment I use to check my blood sugars is convenient; I feel the equipment I use to check my blood sugars is reliable; My blood sugar monitoring fits in with  my lifestyle. | 7-point scale |
| Johnson [67] | Educational and reminder messages | 1) The messages helped me to remember to take my medication  2) The messages helped me to remember to take my blood sugars  3) The messages helped me to eat healthier  4) The messages helped me to become more active  5) I would use the messages in my next pregnancy if I am diagnosed with gestational diabetes.  6) I would recommend the program to a friend with diabetes in pregnancy.  7) Message came at the right time of day  …………………  Which types of messages were more helpful (mark all that apply):  Direct reminders to take medications and blood sugars; Messages regarding healthy eating, Messages regarding being active; Messages regarding complications of diabetes in pregnancy  …………………..  Did you find the number of daily messages: Just right; Too many ; Too few  …………………………  Messages likes (things you liked about messages: Nothing; Content; Reminders  …………………………..  Messages dislikes (things you did not like about messages): Nothing; Content; Frequency; Timing; Lack of personal interaction  …………………………….  Number of text messages read  All; Most; Some; None.  How well did the messages fit into your personal treatment plan?  Fit very well; Fit ok; Did not fit well. | 5-point scale  …………………  percentage |
| Mackillop [43] | Blood glucose management and GDM management messages. | Women’s overall satisfaction with  gestational diabetes care:  I am satisfied with my current treatment; I am satisfied the treatment; I am receiving is the best  for me; I am satisfied with my understanding of diabetes  Relationship with the diabetes clinical care team:  I feel my maternity diabetes team knows enough about my current level of diabetes control; I feel I have a good relationship with my maternity diabetes team; I am satisfied with my maternity diabetes team’s understanding of my diabetes.  Satisfaction with the GDm-health system:  I find the equipment I use to check my blood sugars is convenient; I feel the equipment I use to check my blood sugars is reliable; My blood sugar monitoring fits in with  my lifestyle | 7-point scale |
| Peleg [69] | Blood glucose, ketonuria, diet, blood pressure, and physical activity management; GDM management recommendations. | Patient :  1) I like the fact that the system can adapt to my daily life and context changes;  2) I think that using the app has NOT complicated my daily life;  3) I would recommend the system to other GDM patients;  4) Would you recommend the system to a friend of yours? Y/N  5) Would you use MobiGuide again? Y/N  6) Would you pay for MobiGuide? Y/N  Care provider:  1) Positive addition to patient; 2) Positive addition to our organization; 3) Important part of staffing 4) MobiGuide makes it easier to manage patients; 5) MobiGuide makes it quicker to manage patients; 6) Using MobiGuide I am likely to care better for my patients; 7) MobiGuide is useful for managing patients; 8) MobiGuide presents a more equitable management process; 9) I am satisfied with MobiGuide’s support for patient management; 10) I manage patients in a timely manner using MobiGuide; 11) MobiGuide increases my overall productivity; 12) I’m able to identify priorities using MobiGuide; 13) MobiGuide’s data quality-aware features increases the safety of outpatients. | 5-point scale |
| Peleg [70] | Blood glucose, ketonuria, diet, blood pressure, and physical activity management, GDM management reminders and recommendations. | .  1) App is interesting  2) App is easy to use  3) Sequence of activities  is clear  4) Application response  time  5) Errors were experienced  6) Ease of learning curve  Usefulness  7) System increased patients' confidence  8) Effective visualization  9) App did/did not complicate patients' lives*  10) I like the system's ability to adapt to context  11) Recommending the system to other patients  12) Recommending the system to a friend  13) Continue to use the system  14) Paying for the system | 5-point scale |
| Varnfield [71] | Blood glucose, diet, exercise, weight management; recording symptoms and providing educational materials. | 1) The M♡THer GDM app was helpful in recording my BGLs,  2) I feel confident that my health care team checked my BGLs,  3) The M♡THer GDM app helped me to feel confident  in managing my gestational diabetes,  4) I felt supported by the health care team that was monitoring the M♡THer portal,  5) Overall, I was satisfied with the M♡THer GDM app. | 5-point scale |

The references are consistent with the article’s references.

25. Given JE, Bunting BP, O'Kane MJ, Dunne F, Coates VE. Tele-Mum: a feasibility study for a randomized controlled trial exploring the potential for telemedicine in the diabetes care of those with gestational diabetes. Diabetes Technol Ther 2015 Dec;17(12):880-888. [doi: 10.1089/dia.2015.0147] [Medline: 26394017]

30. Hirst JE, Mackillop L, Loerup L, Kevat DA, Bartlett K, Gibson O, et al. Acceptability and user satisfaction of a smartphone-based, interactive blood glucose management system in women with gestational diabetes mellitus. J Diabetes Sci Technol 2015 Jan;9(1):111-115 [FREE Full text] [doi: 10.1177/1932296814556506] [Medline: 25361643]

34. Caballero-Ruiz E, García-Sáez G, Rigla M, Villaplana M, Pons B, Hernando ME. A web-based clinical decision support system for gestational diabetes: automatic diet prescription and detection of insulin needs. Int J Med Inform 2017 Jun;102:35-49. [doi: 10.1016/j.ijmedinf.2017.02.014] [Medline: 28495347]

43. Mackillop L, Hirst JE, Bartlett KJ, Birks JS, Clifton L, Farmer AJ, et al. Comparing the efficacy of a mobile phone-based blood glucose management system with standard clinic care in women with gestational diabetes: randomized controlled trial. JMIR Mhealth Uhealth 2018 Mar 20;6(3):e71 [FREE Full text] [doi: 10.2196/mhealth.9512] [Medline: 29559428]

50. Bartholomew ML, Soules K, Church K, Shaha S, Burlingame J, Graham G, et al. Managing diabetes in pregnancy using cell phone/internet technology. Clin Diabetes 2015 Oct;33(4):169-174 [FREE Full text] [doi: 10.2337/diaclin.33.4.169] [Medline: 26487790]

67. Johnson QB, Berry DC. Impacting diabetes self-management in women with gestational diabetes mellitus using short messaging reminders. J Am Assoc Nurse Pract 2018 Jun;30(6):320-326. [doi: 10.1097/JXX.0000000000000059] [Medline: 29878964]

69. Peleg M, Shahar Y, Quaglini S, Broens T, Budasu R, Fung N, et al. Assessment of a personalized and distributed patient guidance system. Int J Med Inform 2017 May;101:108-130. [doi: 10.1016/j.ijmedinf.2017.02.010] [Medline: 28347441]

70. Peleg M, Shahar Y, Quaglini S, Fux A, García-Sáez G, Goldstein A, et al. MobiGuide: a personalized and patient-centric decision-support system and its evaluation in the atrial fibrillation and gestational diabetes domains. User Model User-Adap Inter 2017 Mar 11;27(2):159-213. [doi: 10.1007/s11257-017-9190-5]

71. Varnfield M, Redd C, Stoney RM, Higgins L, Scolari N, Warwick R, et al. M♡THer, an mHealth system to support women with gestational diabetes mellitus: feasibility and acceptability study. Diabetes Technol Ther 2021 May;23(5):358-366 [FREE Full text] [doi: 10.1089/dia.2020.0509] [Medline: 33210954]
